# Supplementary material for: Complex temporal dynamics of phage-bacteria populations in an animal-associated marine system
Source: Nat Commun. 2026 Apr 4;17:4870. doi: 10.1038/s41467-026-71398-9 (PMC13230959; doi:10.1038/s41467-026-71398-9)
Supplement: Supplementary file 1 — Supplementary Information [file 41467_2026_71398_MOESM1_ESM.pdf]

Complex temporal dynamics of phage-bacteria populations in an animal-associated marine system

Jeffrey Liang[1§], Karine Cahier[2§], Damien Piel[2], Dario Cueva Granda[1], David Goudenège[2,3], Yannick Labreuche[2,3], Laurence Ma[4], Marc Monot[4], Charles Bernard[5], Eduardo P.C. Rocha[5#] and Frédérique Le Roux[1#]

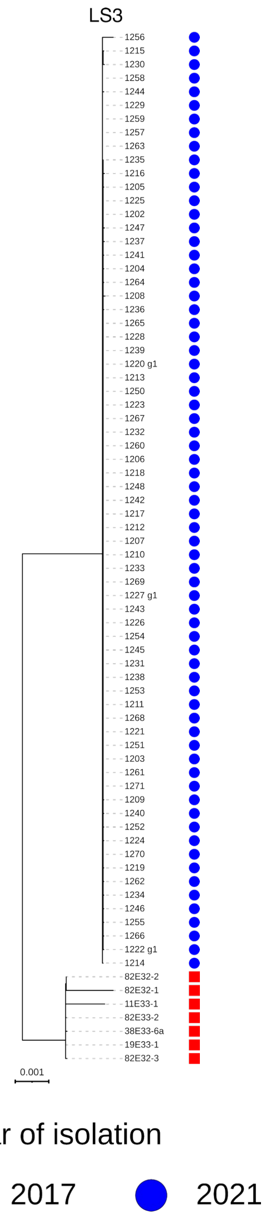

Figure S1. The inferred phylogeny of virulent phages from species LS3 isolated in both 2017 and 2021. Maximum-likelihood trees were generated from whole genome alignments of sequenced phage genomes and are displayed as midpoint-rooted. Shapes to the right annotate the year of isolation.

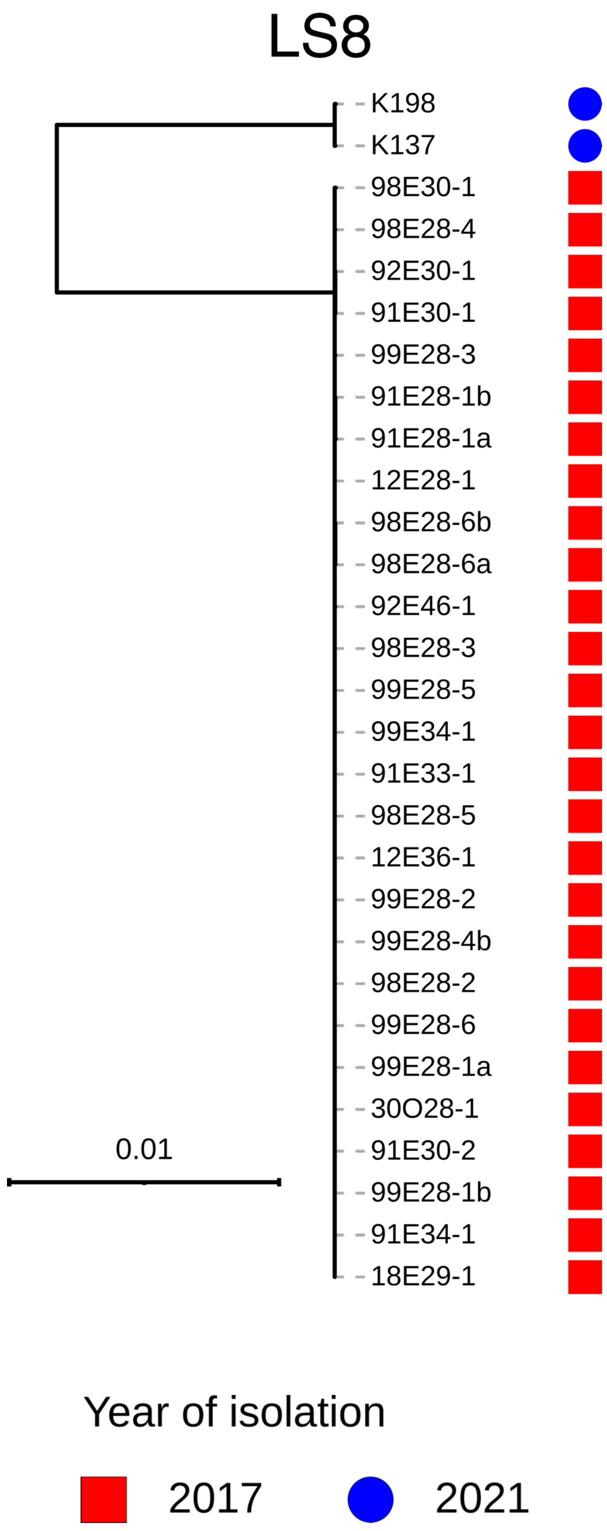

Figure S2. The inferred phylogeny of virulent phages from species LS8 isolated in both 2017 and 2021. Maximum-likelihood trees were generated from whole genome alignments of sequenced phage genomes and are displayed as midpoint-rooted. Shapes to the right annotate the year of isolation.

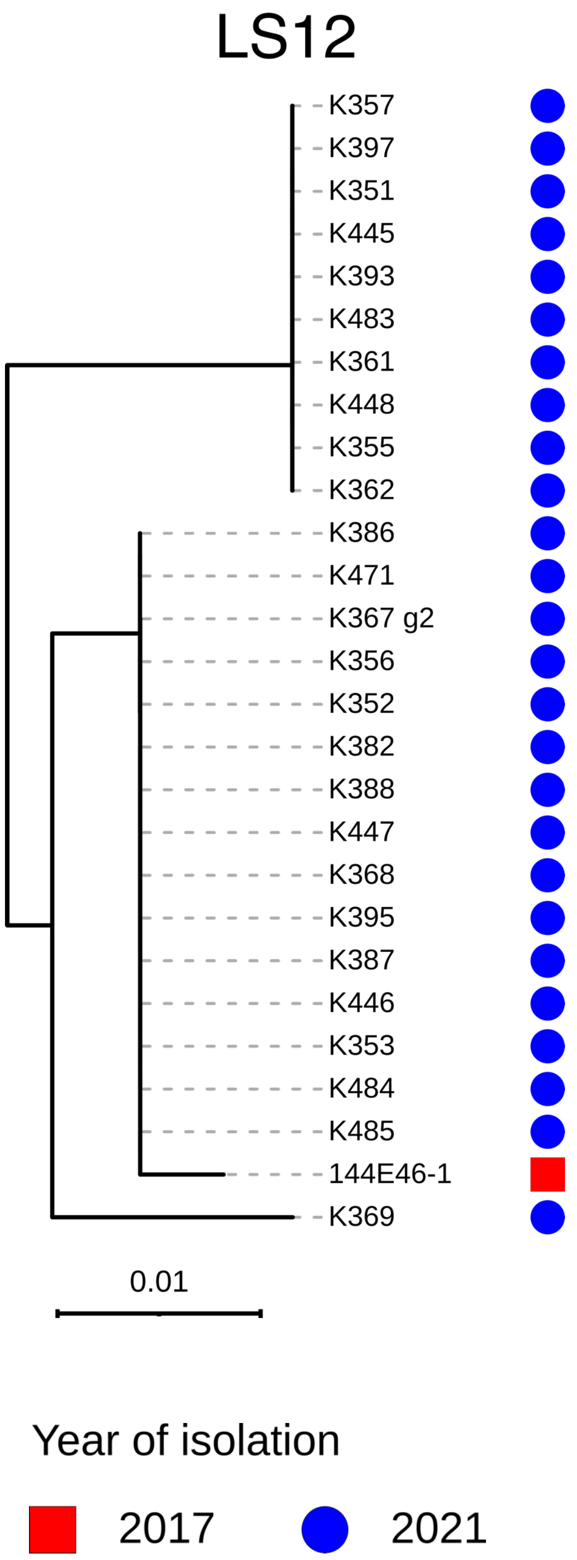

Figure S3. The inferred phylogeny of virulent phages from species LS12 isolated in both 2017 and 2021. Maximum-likelihood trees were generated from whole genome alignments of sequenced phage genomes and are displayed as midpoint-rooted. Shapes to the right annotate the year of isolation.

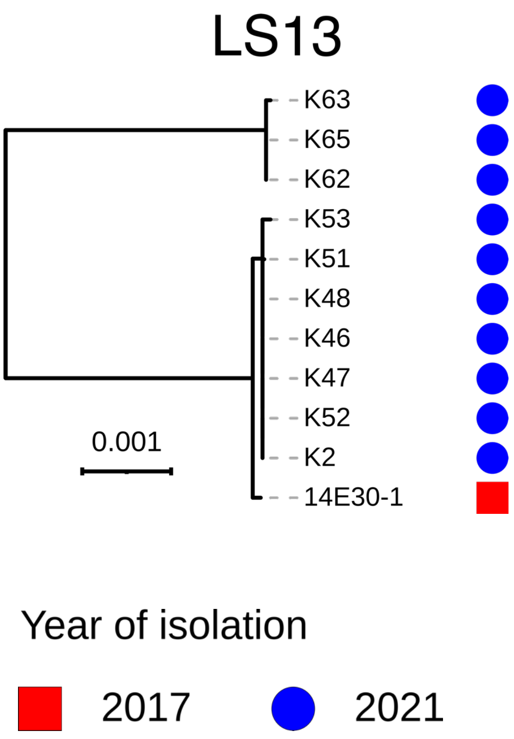

Figure S4. The inferred phylogeny of virulent phages from species LS13 isolated in both 2017 and 2021. Maximum-likelihood trees were generated from whole genome alignments of sequenced phage genomes and are displayed as midpoint-rooted. Shapes to the right annotate the year of isolation.

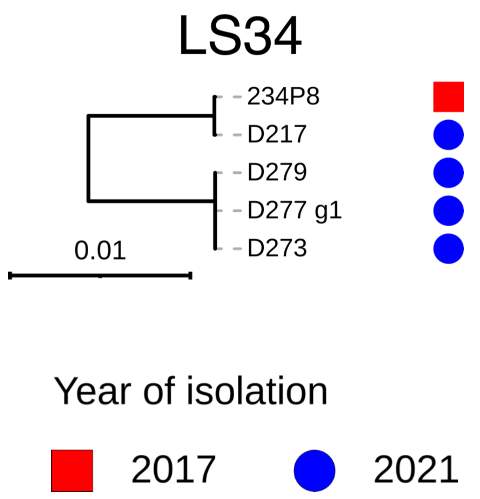

Figure S5. The inferred phylogeny of virulent phages from species LS34 isolated in both 2017 and 2021. Maximum-likelihood trees were generated from whole genome alignments of sequenced phage genomes and are displayed as midpoint-rooted. Shapes to the right annotate the year of isolation.

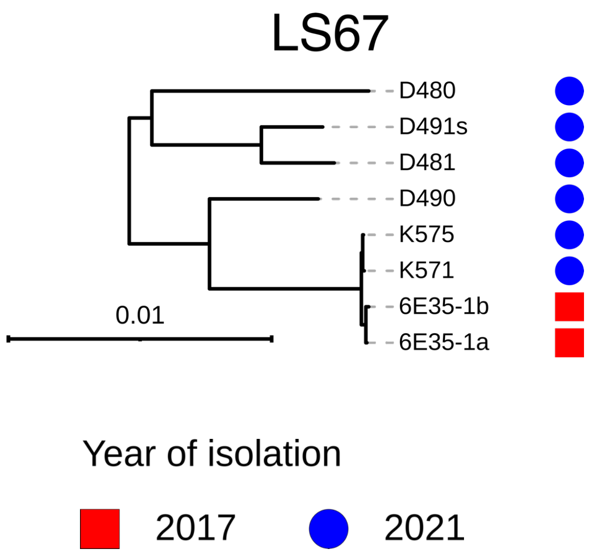

Figure S6. The inferred phylogeny of virulent phages from species LS67 isolated in both 2017 and 2021. Maximum-likelihood trees were generated from whole genome alignments of sequenced phage genomes and are displayed as midpoint-rooted. Shapes to the right annotate the year of isolation.

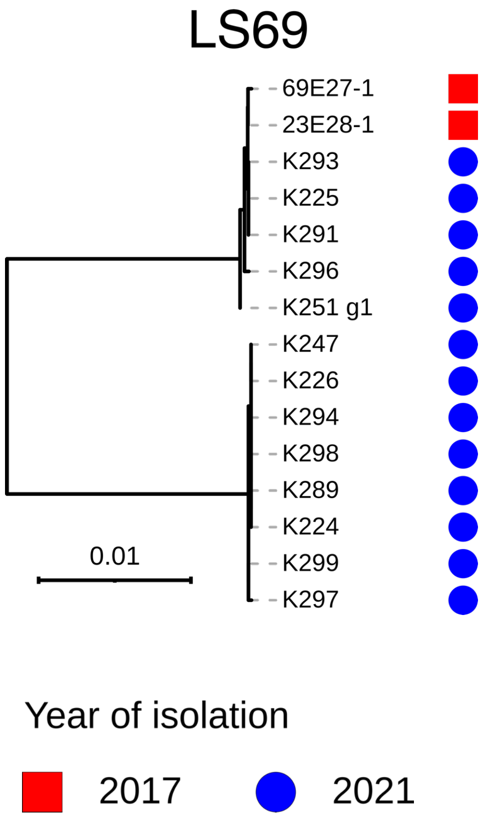

Figure S7. The inferred phylogeny of virulent phages from species LS69 isolated in both 2017 and 2021. Maximum-likelihood trees were generated from whole genome alignments of sequenced phage genomes and are displayed as midpoint-rooted. Shapes to the right annotate the year of isolation.

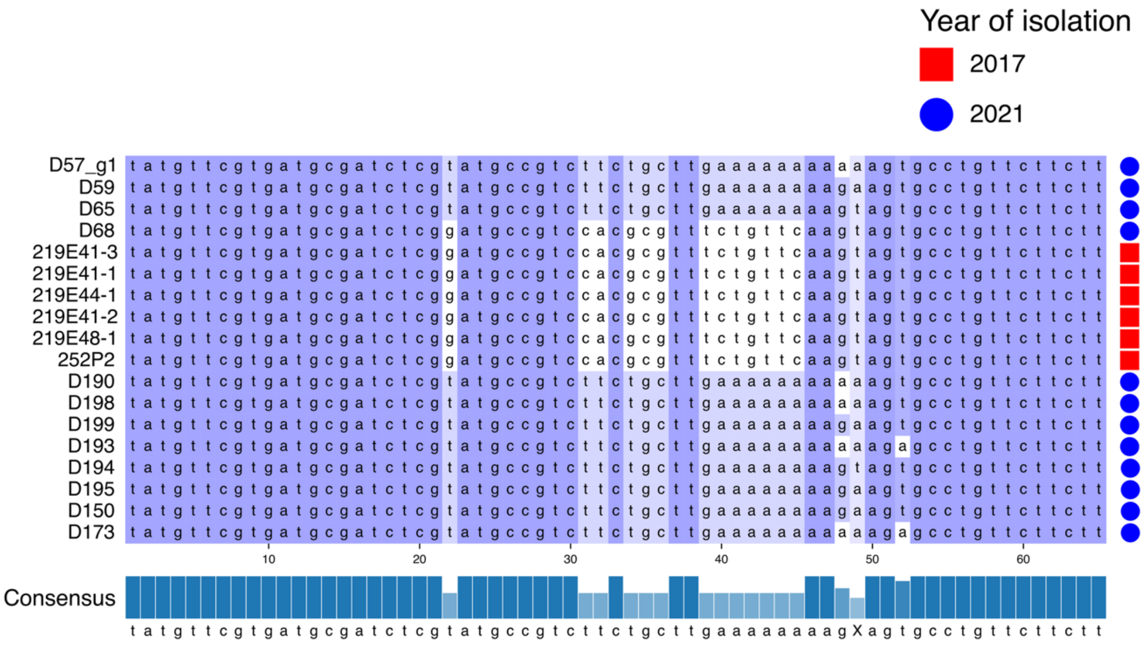

Figure S8. Intergenomic variation in LS29 is largely localized to a short region within a gene of unknown function. A section of a whole genome alignment between 18 phages of LS29 shows 16 variable sites which are a major component of the phylogenetic distance separating 2021 phage isolates from 2017 phages (and D68). The year of isolation of each phage is indicated to the right. Sequences are colored by percent conservation at each site and the consensus sequence and degree of conservation are shown in the bottom bar.

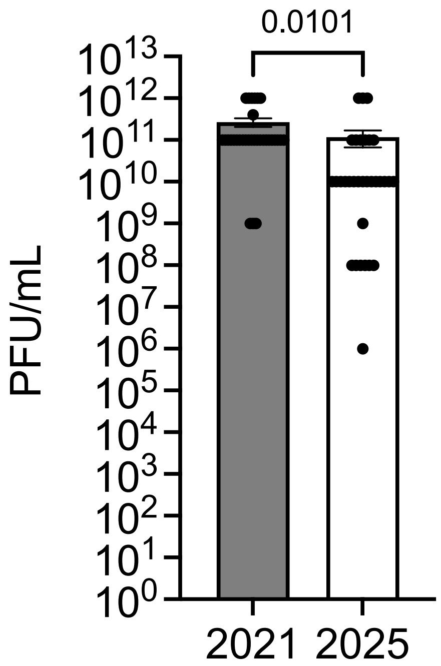

Figure S9. Phage stability *in vitro*. Phage isolates retained viability after four years, with titers decreasing on average by approximately one log unit. A total of 32 randomly selected lytic phages (individual data points are shown using overlaid dot plots) were analyzed. Statistical significance was assessed using a paired *t*-test.

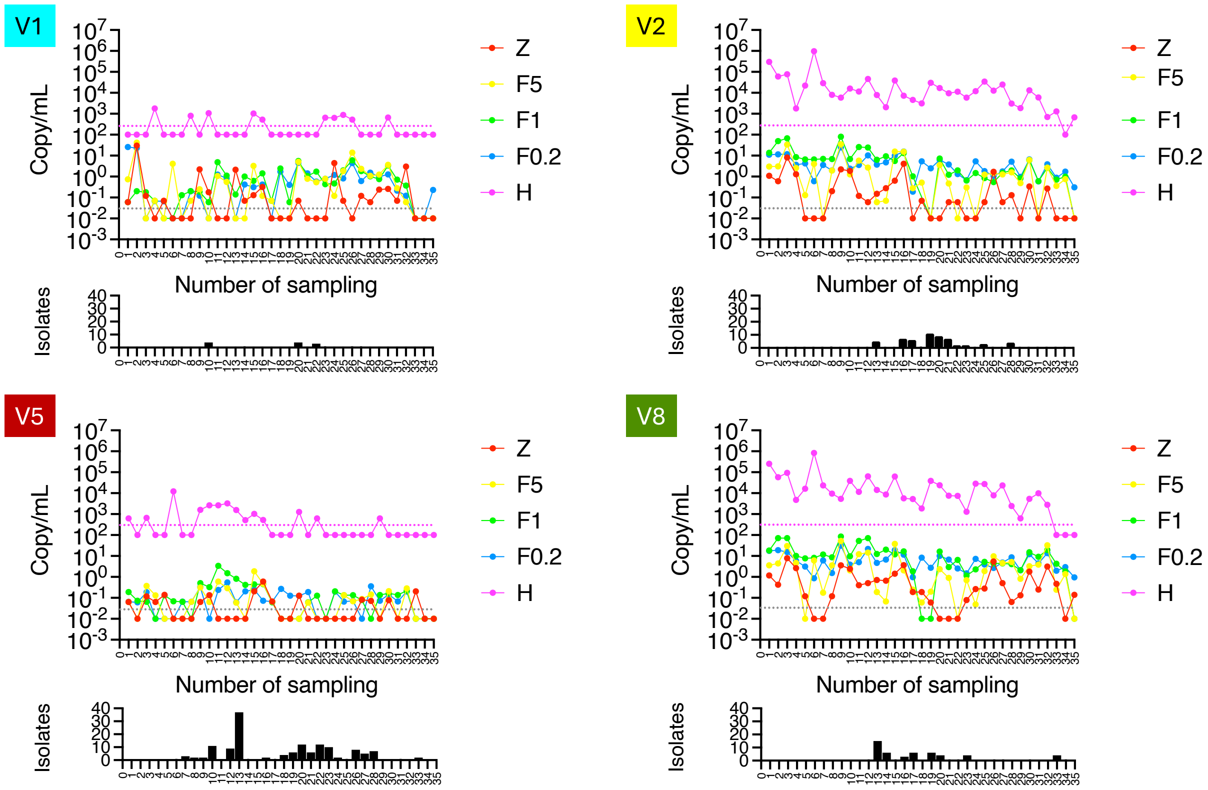

Figure S10. Quantification of *V. crassostreae* clades in seawater and oyster hemolymph by digital droplet PCR (ddPCR). Seawater was size-fractionated (Z>60 mm; F5: 60-5 mm; F1: 5-1 mm; F0.2: 1-0.2 mm; H: hemolymph) and DNA extracted from each fraction, while hemolymph from 90 oysters was pooled for DNA extraction. Each point represents the absolute DNA copy number per mL of hemolymph or seawater. Each experiment was performed once. The dotted horizontal lines indicate the limits of quantification, set at 1 copy per 20 µL reaction and adjusted to account for sample dilution. Histograms below each graph show the number of strains isolated per date. Source data are provided as a Source Data file.

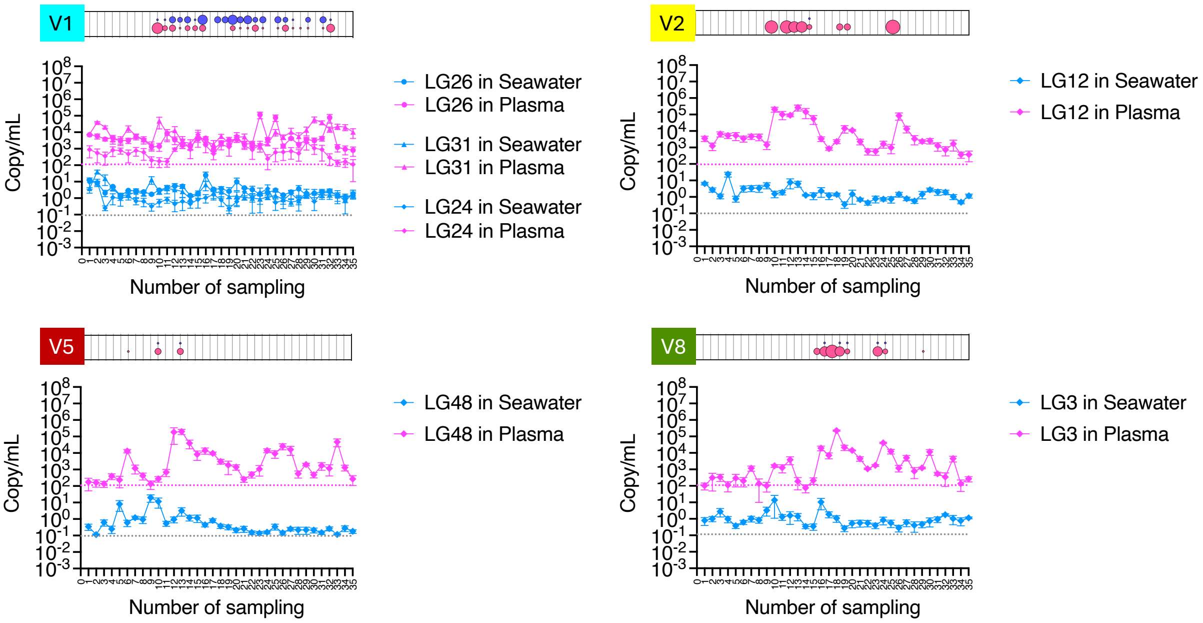

Figure S11. Quantification of phage genera in seawater and oyster plasma by digital droplet PCR (ddPCR). Viral fractions from seawater (<0.2 µm) were concentrated 1,000-fold using iron chloride flocculation, while viral fractions from plasma (<0.2 µm) were obtained from pooled hemolymph of 90 oysters. Viral DNA was extracted and quantified by ddPCR. ddPCR was performed four times by two experimenters and using Evagreen or multiplexed probes. Each point represents the absolute DNA copy number per mL of plasma or seawater. The dotted horizontal lines indicate the limits of quantification. Dot plots above each graph show average PFU counts per clade and sampling date, measured from the same samples (10 µL of seawater concentrate, equivalent to 10 mL of raw seawater, or 10 µL of oyster plasma; see Figure 3A), providing an independent validation of the ddPCR data. Source data are provided as a Source Data file.

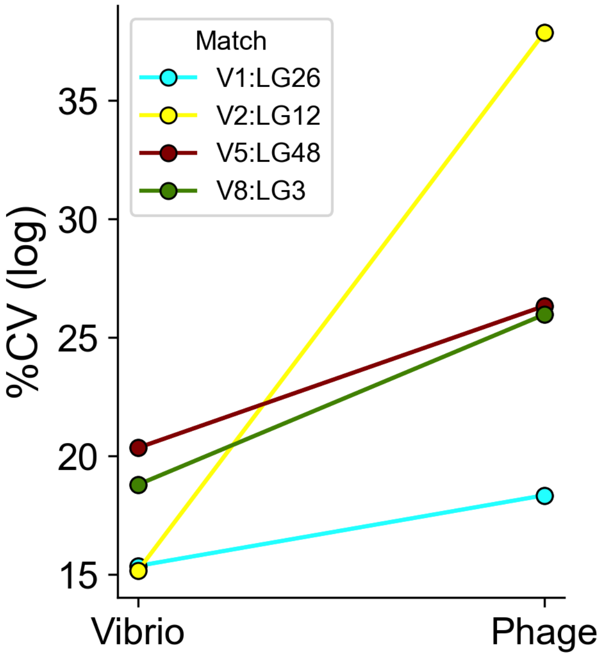

Figure S12. Coefficients of variation of the abundance of vibrio and phages.
Connections link each phage to the bacterial clade of its host tropism. The values are log10 transformed ddPCR DNA quantification. The coefficient of variation is the ratio between the standard deviation and the mean and was computed across the 35 sampling dates for each time series.

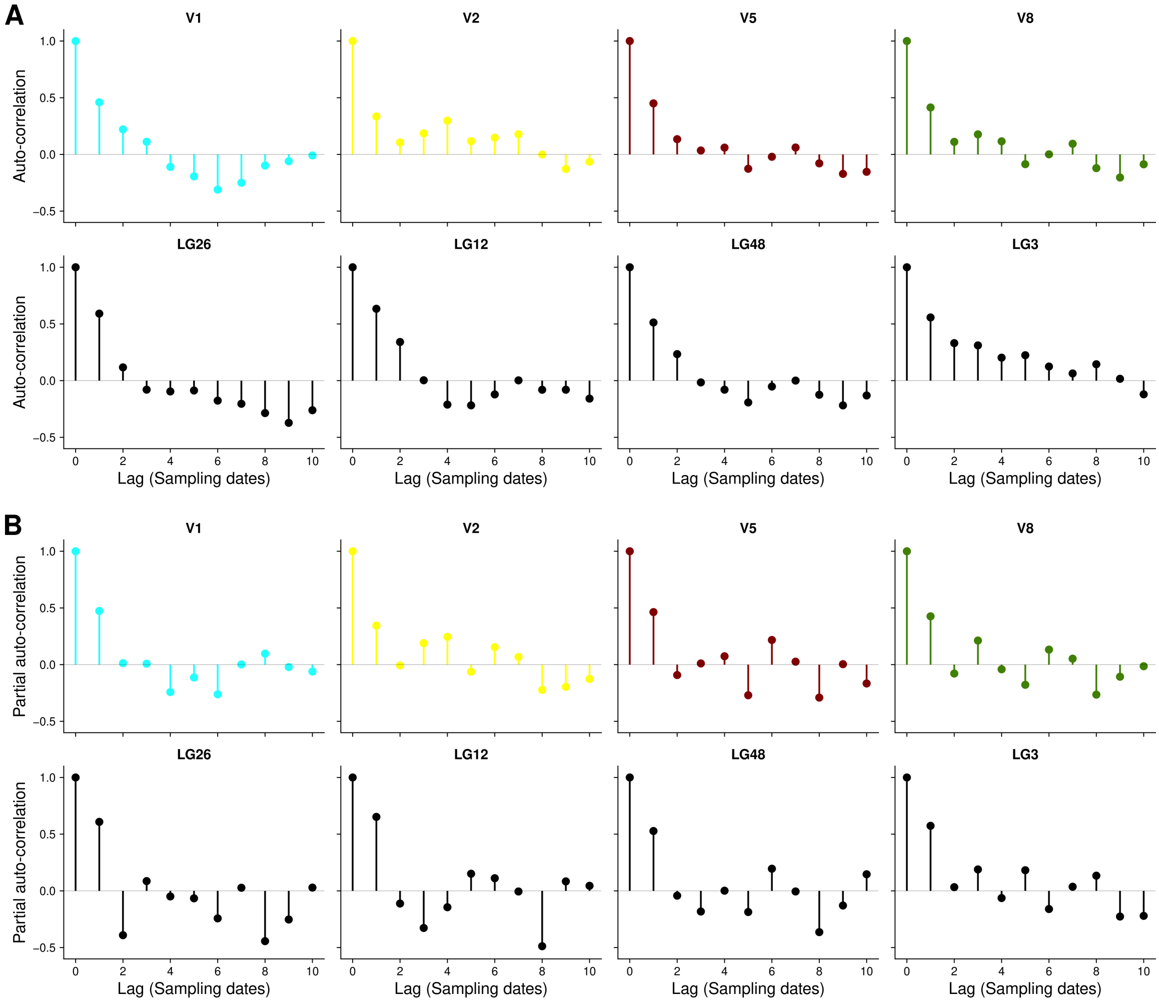

Figure S13. Auto-correlation and partial auto-correlations of the time series abundance data. The log10 ddPCR DNA quantifications for each probed *V. crassostreae* clade (top rows) or phage genus (bottom rows) were averaged over 10 oysters in each of the 35 sampling dates. **A.** Auto-correlations were calculated out to a maximum lag of 10 sampling dates (21-22 ordinal days). **B.** Partial auto-correlations were calculated out to a maximum lag of 10 sampling dates.

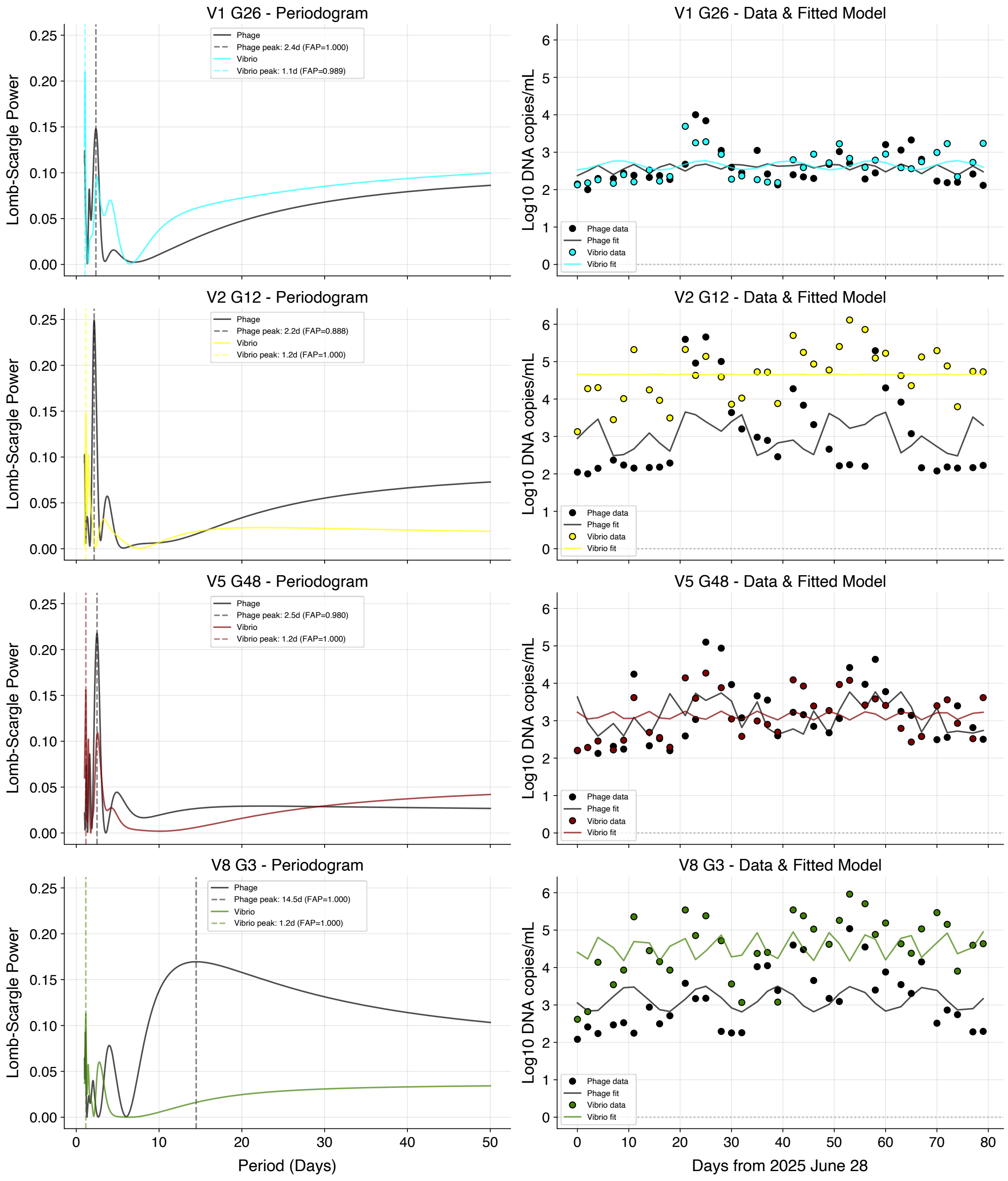

Figure S14. Lomb-Scargle periodograms for the detection of periodicity in the abundance of phages and bacteria populations over the time series. Lomb-Scargle periodograms were computed with the mean log10 ddPCR DNA quantifications for each *V. crassostreae* clade and phage genus using the ordinal dates of each sampling point. Power spectra are plotted in pairs of corresponding bacteria and phages, with the period of peak power density marked by the dashed vertical lines. The poor evidence for periodicity for each time series can be concluded from the observation of the high false alarm probabilities (FAP) which is annotated in periodogram legends. It can also be confirmed from the observation of the fitted models to the right of the figure.

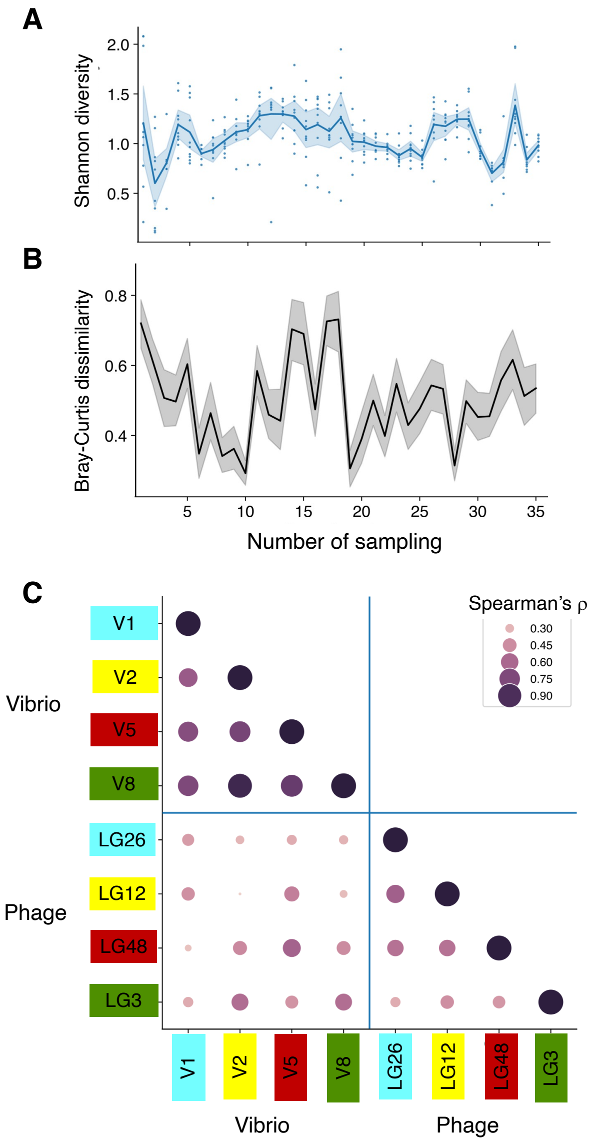

Figure S15. Diversity indices of oysters infected by *V. crassostreae* clades and their corresponding lytic phage genera (as shown in Figure 2), based on ddPCR-determined DNA copy number per mL. A. Alpha-diversity, measured using the Shannon index, shows temporal fluctuations over the sampling period but no clear overall trend. Each point represents an individual oyster; the solid line indicates the mean, with shading denoting the 95% confidence interval. **B.** Beta-diversity**,** calculated as the mean Bray-Curtis dissimilarity across all 45 pairwise comparisons of 10 oysters per date, reflects variability in the composition of vibrio clades or phage genera. Periods of low beta diversity (i.e., more uniform composition across oysters) often coincide with population blooms. Shading shows the 95% confidence interval. **C.** Spearman correlations of ddPCR DNA copy number per mL between tracked populations reveal strong co-fluctuations among vibrio clades but surprisingly weak correlations between each vibrio clade and its corresponding phage genus.

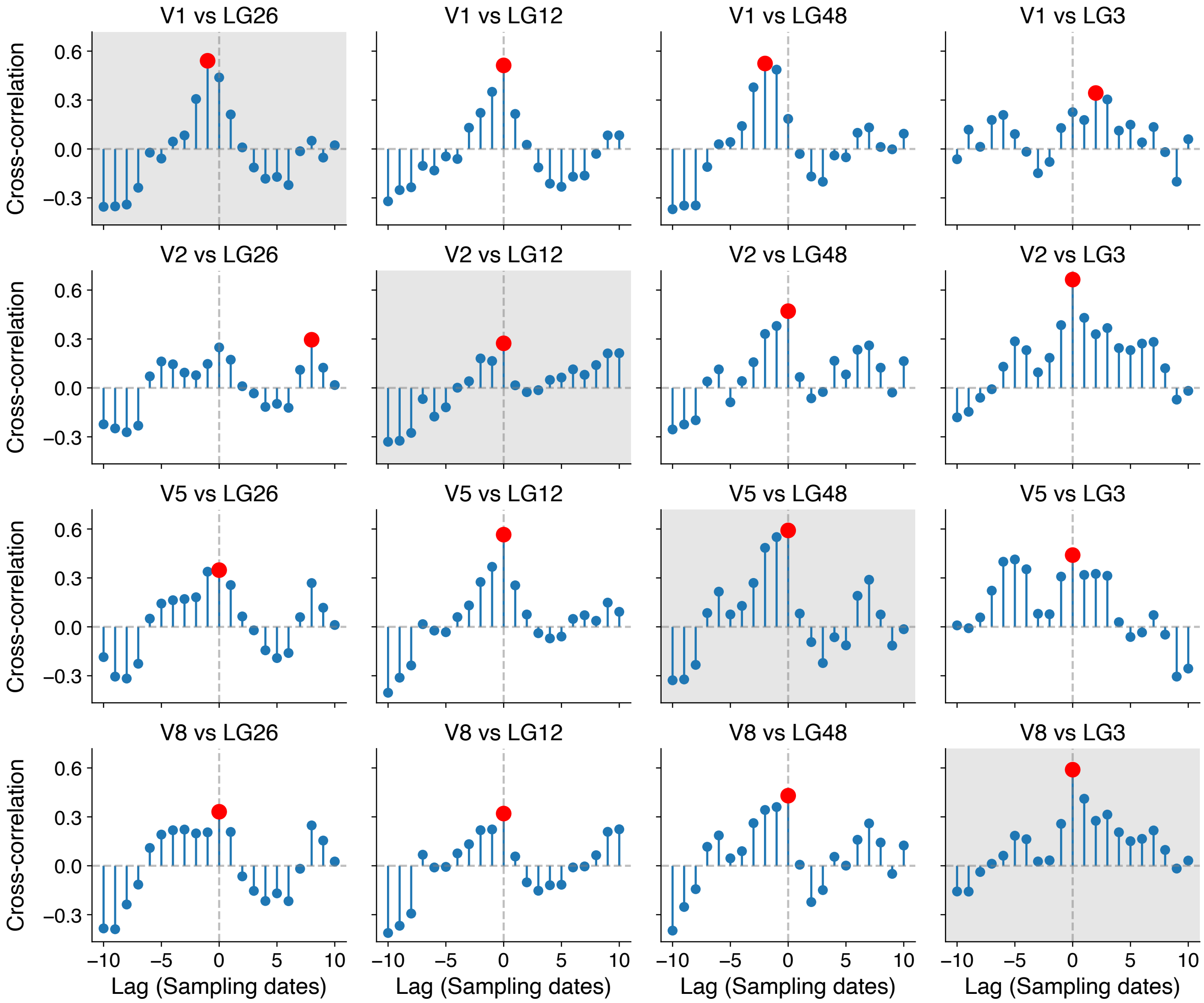

Figure S16. Cross-correlation analysis of the time series abundance data between phage and bacteria populations. Cross-correlations between the mean log10 ddPCR DNA quantifications between each probed *V. crassostreae* clade and phage genus in each of 35 sampling dates. Shaded plots show the known pairings between phages and their targeted bacterial clades. The highest correlation time lag for each pair is highlighted with a red marker. Positive lag times show correlations calculated when the bacterial populations (V) lead the phage populations (LG) in time, and vice-versa negative lag times.

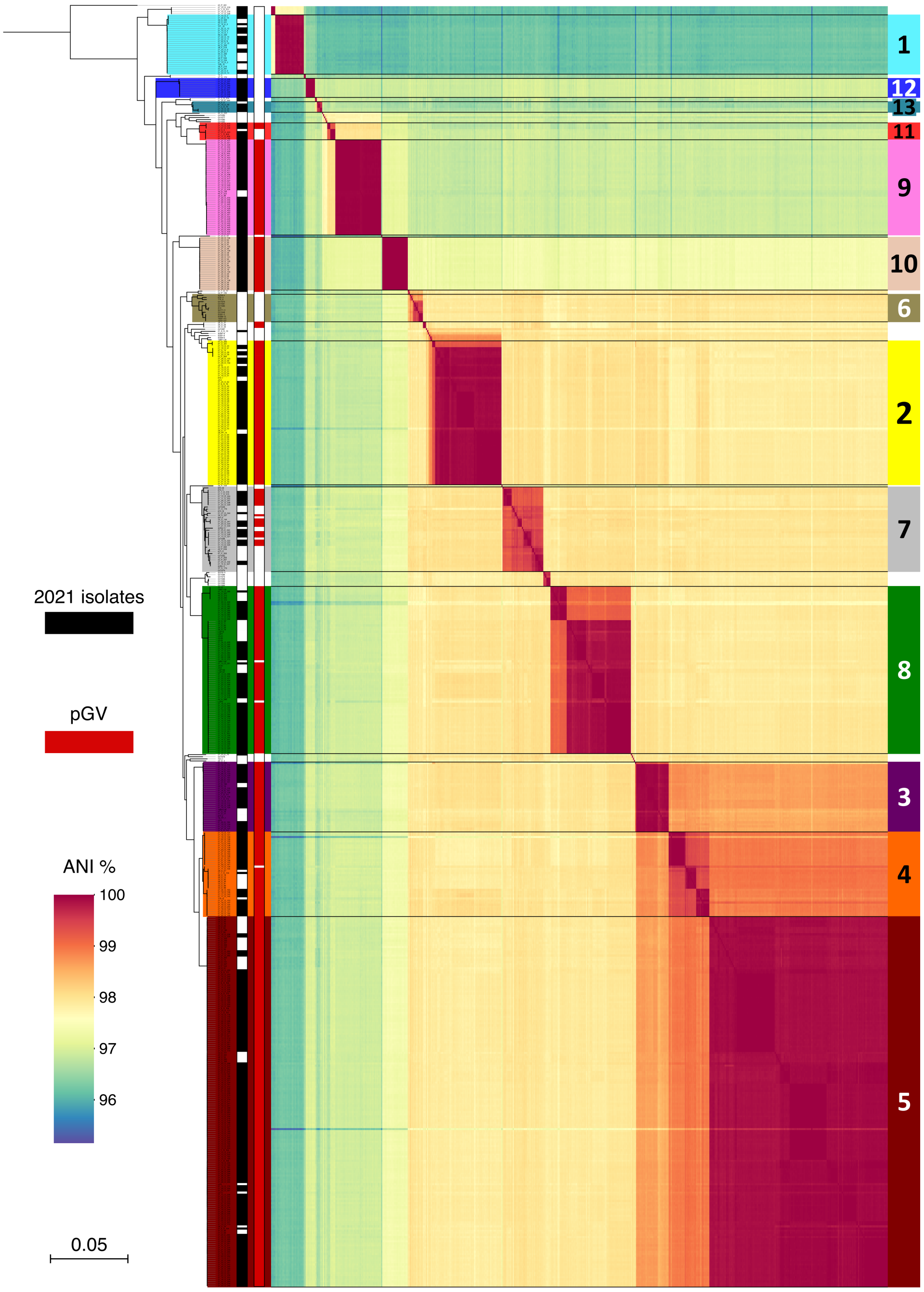

Figure S17. Categorization of bacterial isolates into clades within the *V. crassostreae* species. Core genome phylogeny based on 3,099 gene families of 605 *V. crassostreae* isolates. The pairwise Average Nucleotide Identity (ANI) are plotted in the heatmap, their values revealing clearly distinct clades within the species. Clade designations and corresponding colors refer to[12] with new clades designated V9 to V13. The second column represents sampling, with 2021 dates in black and the other data collections in white. The presence of the virulence-associated plasmid, pGV, is highlighted in red in the second column.

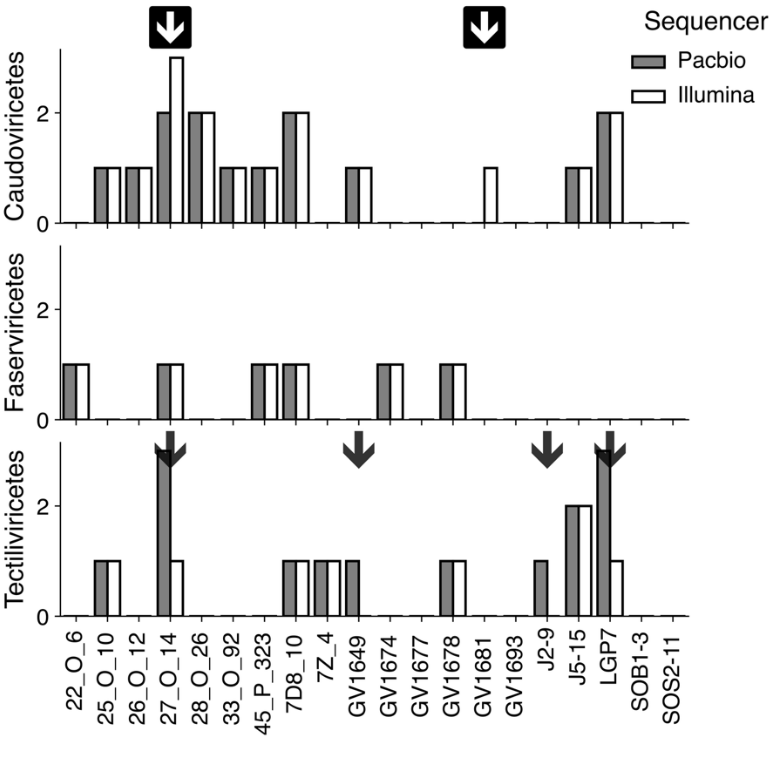

Figure S18. Comparison of prophage detection in 20 *V. crassostreae* genomes sequenced with both PacBio and Illumina platforms. White arrows indicate the detection of *Caudoviricetes* by Illumina sequencing that were not identified by PacBio sequencing. Black arrows indicate the detection of *Tectiliviricetes* by Pacbio sequencing that were not identified by Illumina sequencing**.**

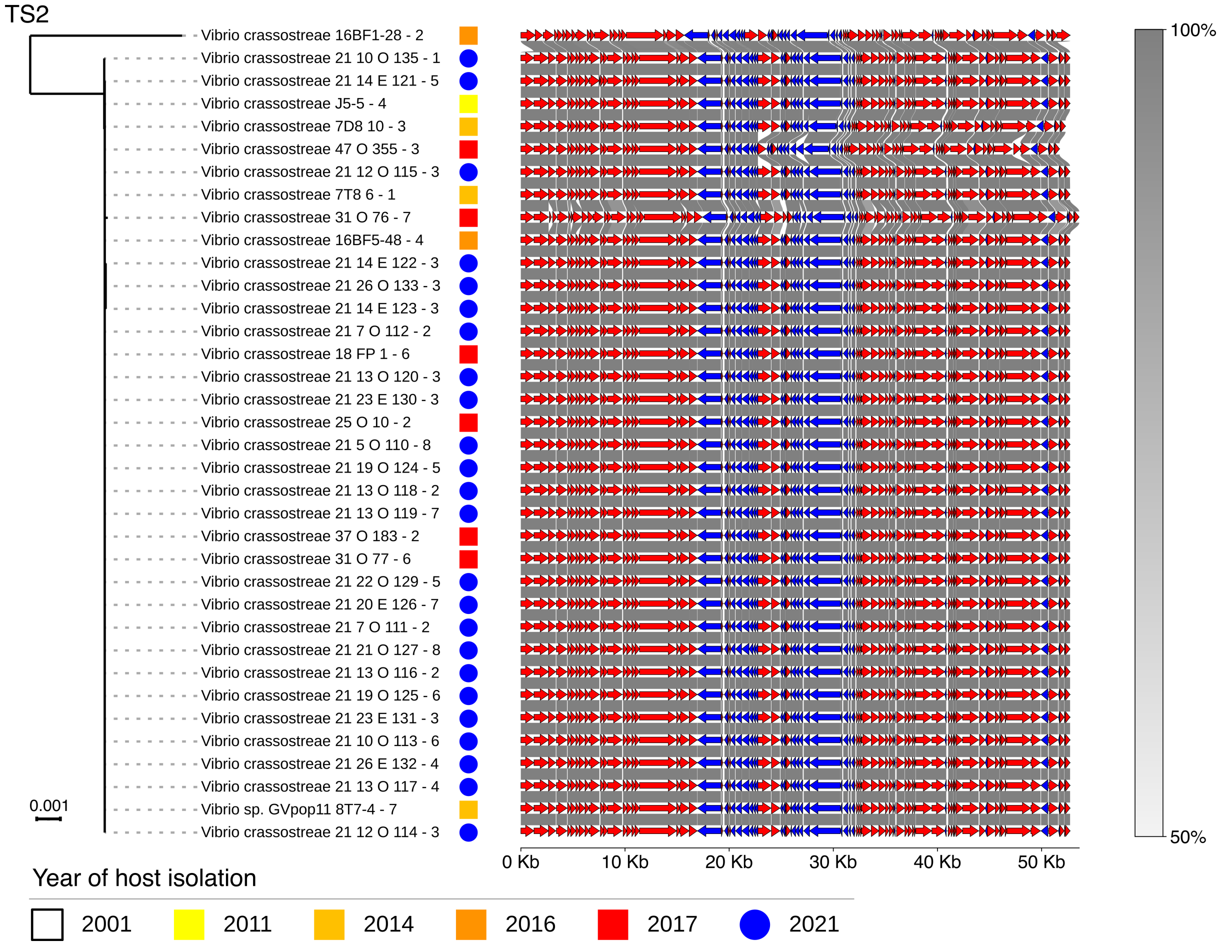

Figure S19. Phylogeny of temperate phage species TS2 identified in *V. crassostreae* strains isolated between 2011 and 2021. The maximum-likelihood tree was generated from whole-genome alignments of phage genomes and is displayed rooted using the midpoint-root method. Annotations to the right indicate the year of isolation for each phage, following the color code shown in the legend. Visualization of synteny shows each temperate phage genome with red and blue arrows showing sense and anti-sense ORFs predicted using pharokka. Links between genes in adjacent genomes show sequence similarity between bidirectional best hits calculated using MMseqs2 and visualized using pygenomeviz.

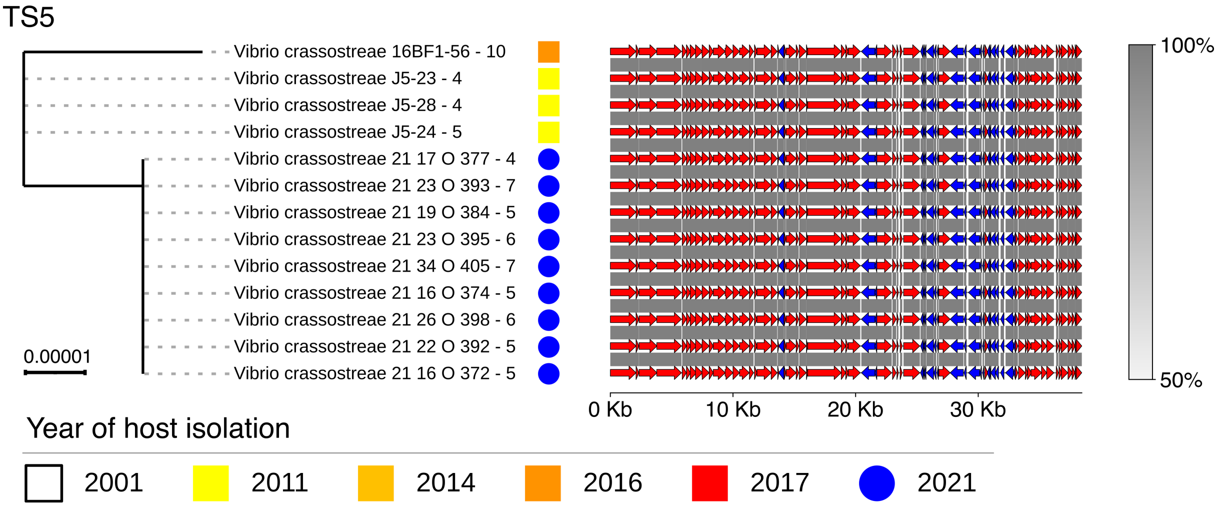

Figure S20. Phylogeny of temperate phage species TS5 identified in *V. crassostreae* strains isolated between 2011 and 2021. Maximum-likelihood trees generated from whole-genome alignments of phage genomes are displayed as midpoint-rooted. Annotations to the right indicate the year of isolation for each phage, following the color code shown in the legend. Visualization of synteny shows each temperate phage genome with red and blue arrows showing sense and anti-sense ORFs predicted using pharokka. Links between genes in adjacent genomes show similarity between bidirectional best hits calculated using MMseqs2 and visualized using pygenomeviz.

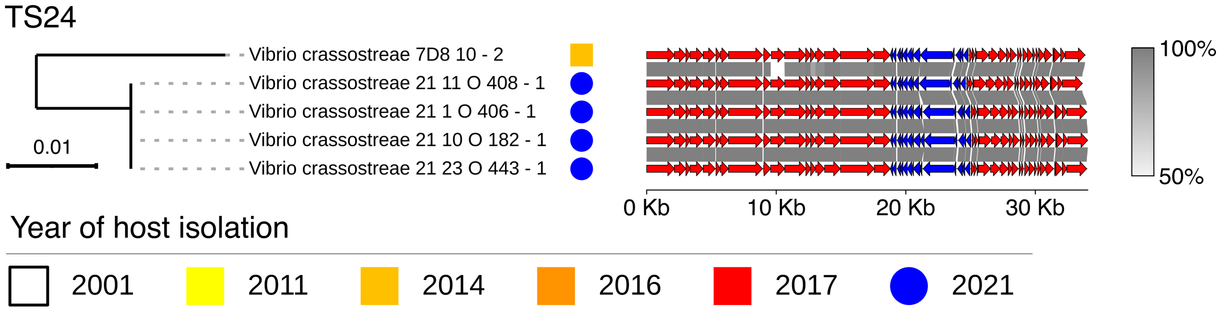

Figure S21. Phylogeny of temperate phage species TS24 identified in *V. crassostreae* strains isolated between 2014 and 2021. Maximum-likelihood trees generated from whole-genome alignments of phage genomes are displayed as midpoint-rooted. Annotations to the right indicate the year of isolation for each phage, following the color code shown in the legend. Visualization of synteny shows each temperate phage genome with red and blue arrows showing sense and anti-sense ORFs predicted using pharokka. Links between genes in adjacent genomes show similarity between bidirectional best hits calculated using MMseqs2 and visualized using pygenomeviz.

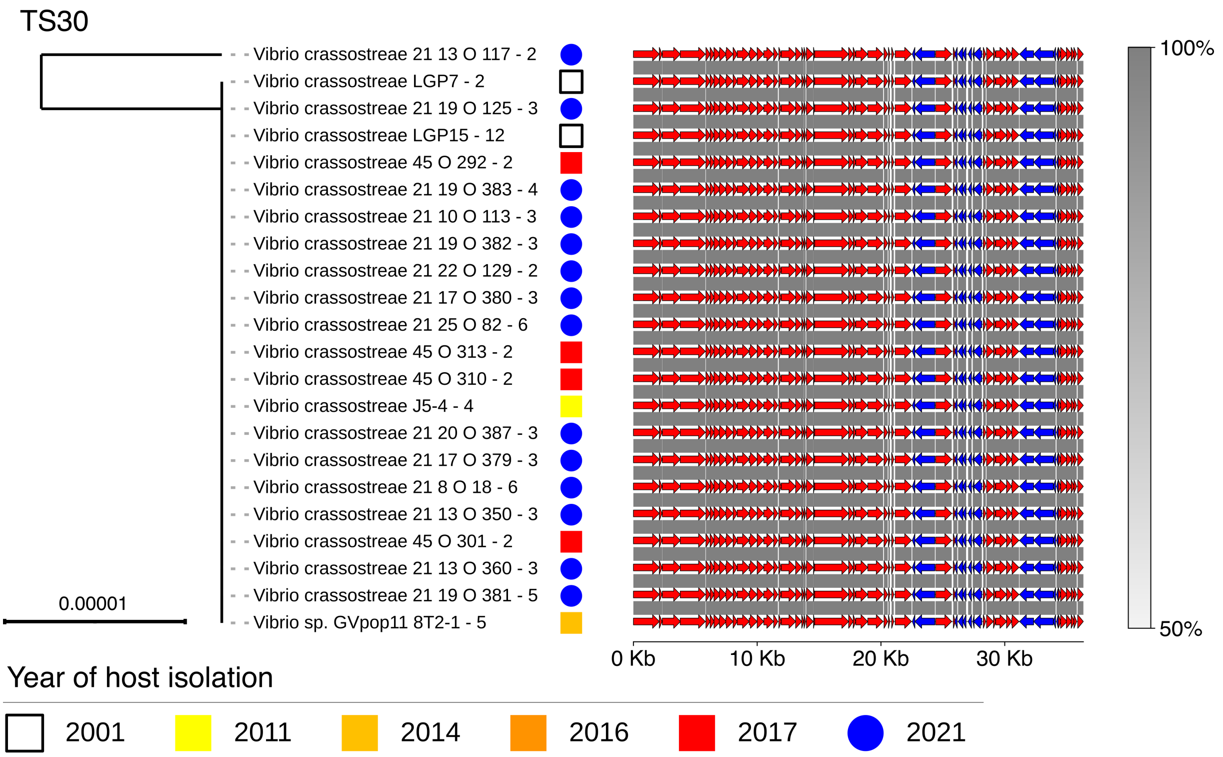

Figure S22. Phylogeny of temperate phage species TS30 identified in *V. crassostreae* strains isolated between 2001 and 2021. Maximum-likelihood trees generated from whole-genome alignments of phage genomes are displayed as midpoint-rooted. Annotations to the right indicate the year of isolation for each phage, following the color code shown in the legend. Visualization of synteny shows each temperate phage genome with red and blue arrows showing sense and anti-sense ORFs predicted using pharokka. Links between genes in adjacent genomes show similarity between bidirectional best hits calculated using MMseqs2 and visualized using pygenomeviz.

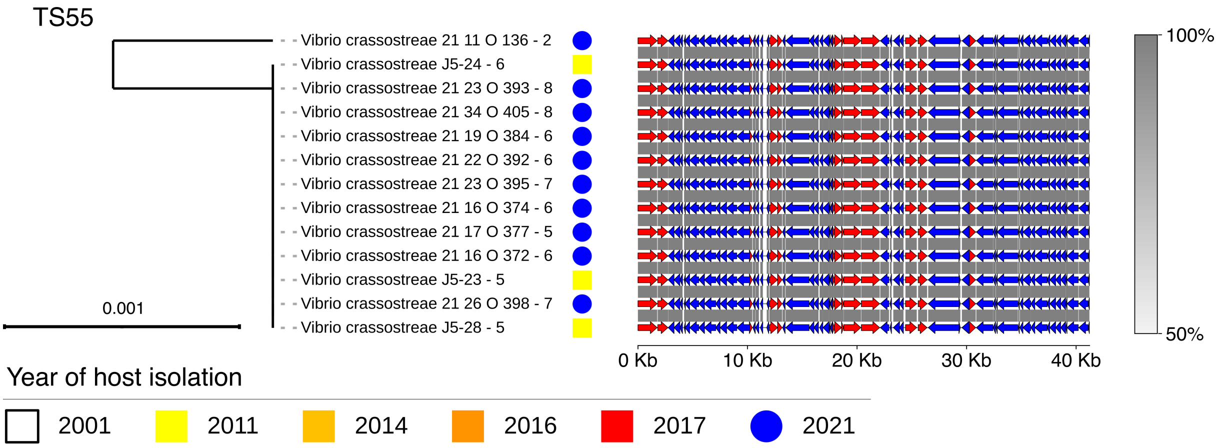

Figure S23. Phylogeny of temperate phage species TS55 identified in *V. crassostreae* strains isolated between 2011 and 2021. Maximum-likelihood trees generated from whole-genome alignments of phage genomes are displayed as midpoint-rooted. Annotations to the right indicate the year of isolation for each phage, following the color code shown in the legend. Visualization of synteny shows each temperate phage genome with red and blue arrows showing sense and anti-sense ORFs predicted using pharokka. Links between genes in adjacent genomes show similarity between bidirectional best hits calculated using MMseqs2 and visualized using pygenomeviz.

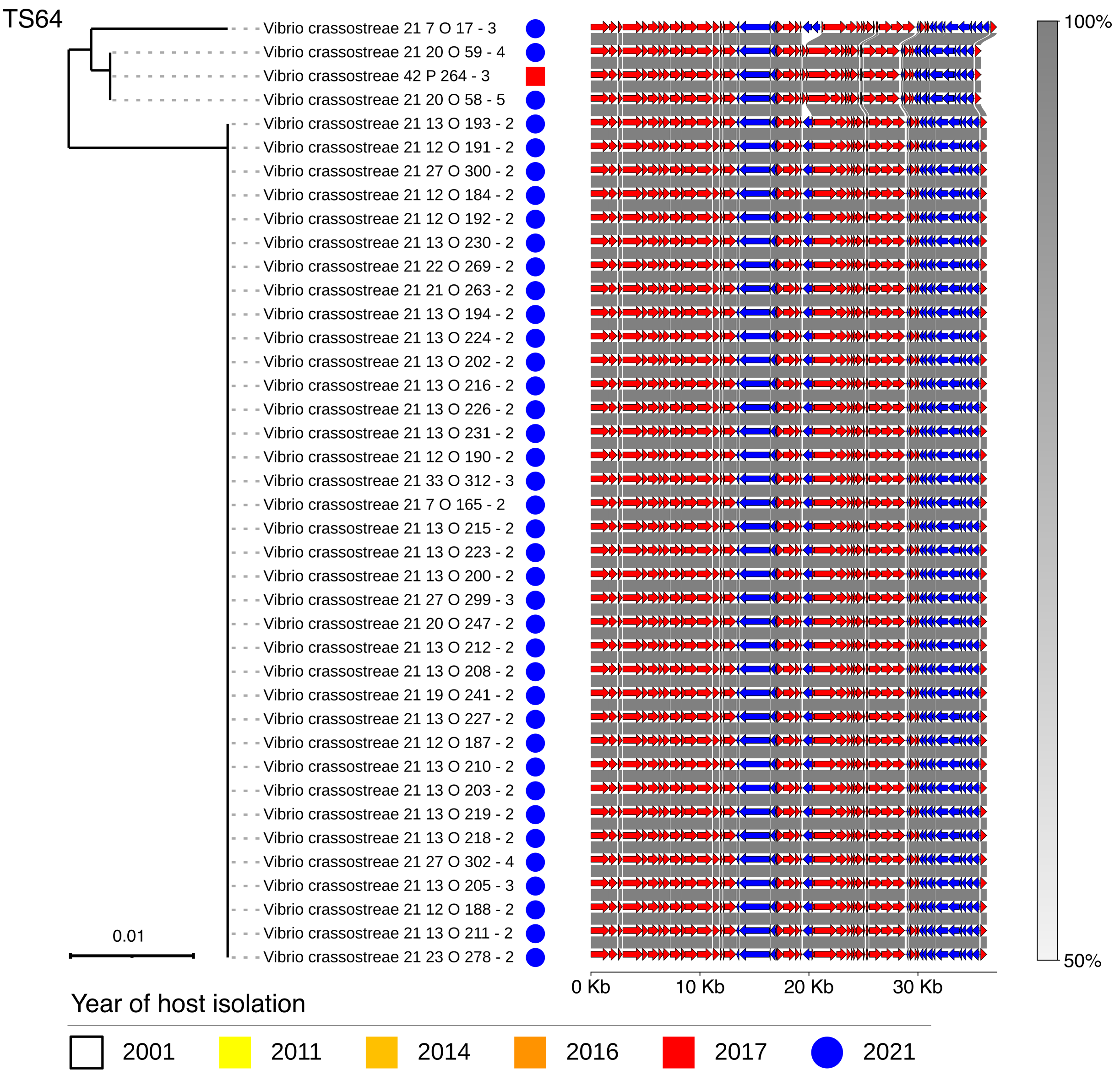

Figure S24. Phylogeny of temperate phage species TS64 identified in *V. crassostreae* strains isolated between 2017 and 2021. Maximum-likelihood trees generated from whole-genome alignments of phage genomes are displayed as midpoint-rooted. Annotations to the right indicate the year of isolation for each phage, following the color code shown in the legend. Visualization of synteny shows each temperate phage genome with red and blue arrows showing sense and anti-sense ORFs predicted using pharokka. Links between genes in adjacent genomes show similarity between bidirectional best hits calculated using MMseqs2 and visualized using pygenomeviz.

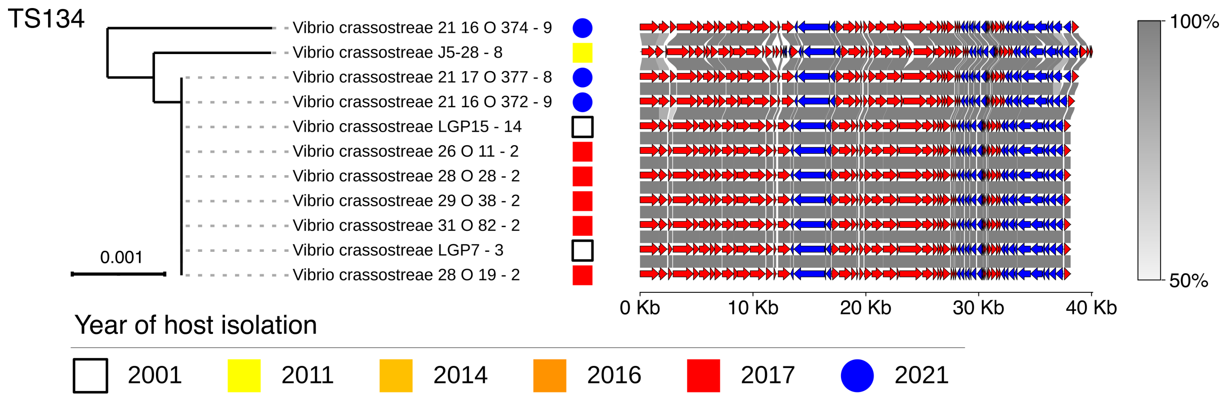

Figure S25. Phylogeny of temperate phage species TS134 identified in *V. crassostreae* strains isolated between 2001 and 2021. Maximum-likelihood trees generated from whole-genome alignments of phage genomes are displayed as midpoint-rooted. Annotations to the right indicate the year of isolation for each phage, following the color code shown in the legend. Visualization of synteny shows each temperate phage genome with red and blue arrows showing sense and anti-sense ORFs predicted using pharokka. Links between genes in adjacent genomes show similarity between bidirectional best hits calculated using MMseqs2 and visualized using pygenomeviz.

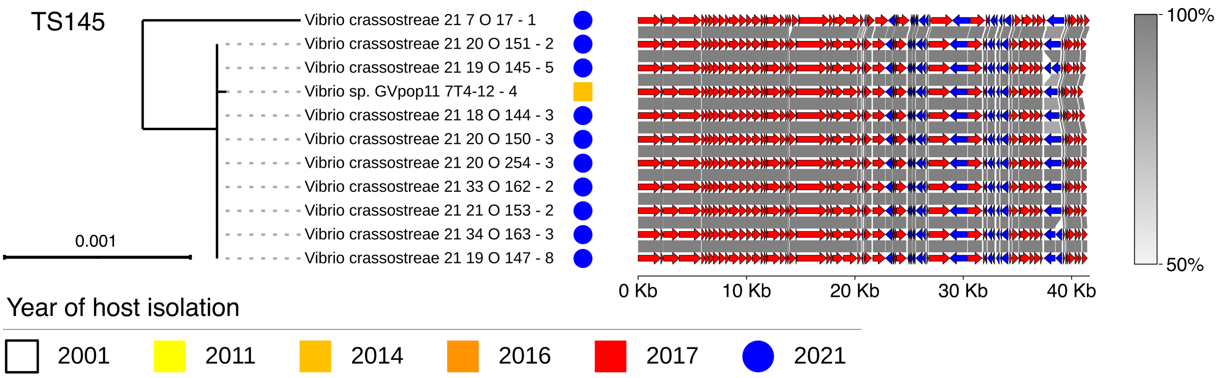

Figure S26. Phylogeny of temperate phage species TS145 identified in *V. crassostreae* strains isolated between 2014 and 2021. Maximum-likelihood trees generated from whole-genome alignments of phage genomes are displayed as midpoint-rooted. Annotations to the right indicate the year of isolation for each phage, following the color code shown in the legend. Visualization of synteny shows each temperate phage genome with red and blue arrows showing sense and anti-sense ORFs predicted using pharokka. Links between genes in adjacent genomes show similarity between bidirectional best hits calculated using MMseqs2 and visualized using pygenomeviz.

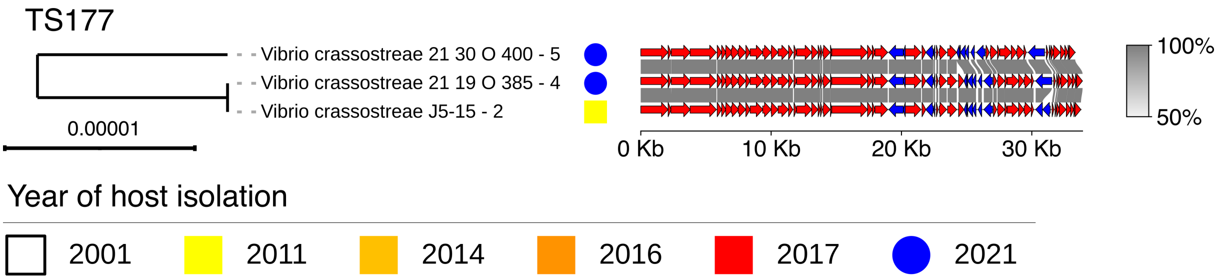

Figure S27. Phylogeny of temperate phage species TS177 identified in *V. crassostreae* strains isolated between 2011 and 2021. Maximum-likelihood trees generated from whole-genome alignments of phage genomes are displayed as midpoint-rooted. Annotations to the right indicate the year of isolation for each phage, following the color code shown in the legend. Visualization of synteny shows each temperate phage genome with red and blue arrows showing sense and anti-sense ORFs predicted using pharokka. Links between genes in adjacent genomes show similarity between bidirectional best hits calculated using MMseqs2 and visualized using pygenomeviz.

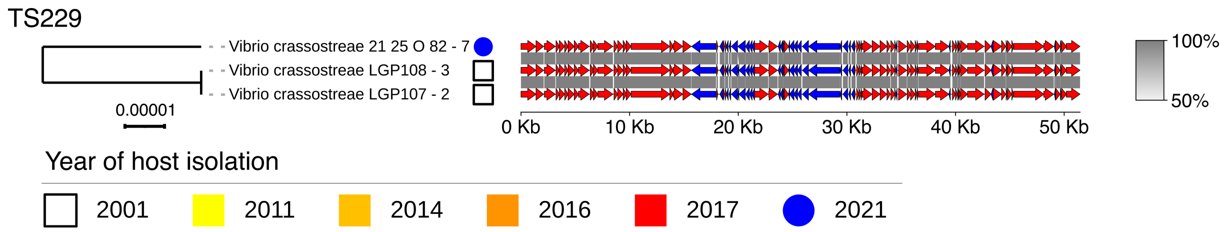

Figure S28. Phylogeny of temperate phage species TS229 identified in *V. crassostreae* strains isolated between 2001 and 2021. Maximum-likelihood trees generated from whole-genome alignments of phage genomes are displayed as midpoint-rooted. Annotations to the right indicate the year of isolation for each phage, following the color code shown in the legend. Visualization of synteny shows each temperate phage genome with red and blue arrows showing sense and anti-sense ORFs predicted using pharokka. Links between genes in adjacent genomes show similarity between bidirectional best hits calculated using MMseqs2 and visualized using pygenomeviz.

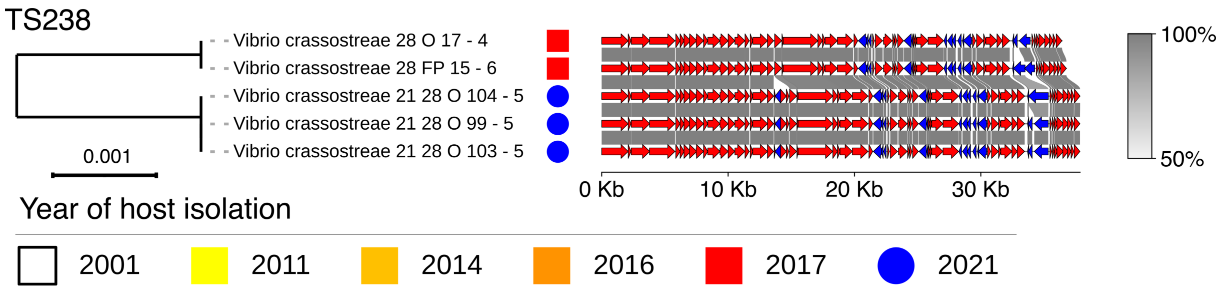

Figure S29. Phylogeny of temperate phage species TS238 identified in *V. crassostreae* strains isolated between 2017 and 2021. Maximum-likelihood trees generated from whole-genome alignments of phage genomes are displayed as midpoint-rooted. Annotations to the right indicate the year of isolation for each phage, following the color code shown in the legend. Visualization of synteny shows each temperate phage genome with red and blue arrows showing sense and anti-sense ORFs predicted using pharokka. Links between genes in adjacent genomes show similarity between bidirectional best hits calculated using MMseqs2 and visualized using pygenomeviz.

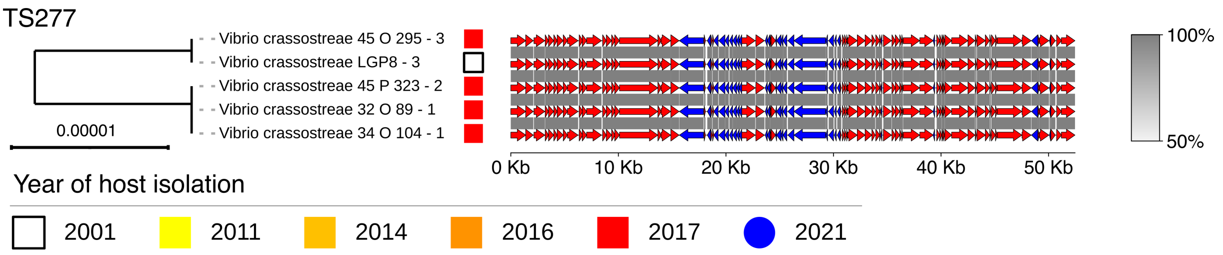

Figure S30. Phylogeny of temperate phage species TS277 identified in *V. crassostreae* strains isolated between 2001 and 2017. Maximum-likelihood trees generated from whole-genome alignments of phage genomes are displayed as midpoint-rooted. Annotations to the right indicate the year of isolation for each phage, following the color code shown in the legend. Visualization of synteny shows each temperate phage genome with red and blue arrows showing sense and anti-sense ORFs predicted using pharokka. Links between genes in adjacent genomes show similarity between bidirectional best hits calculated using MMseqs2 and visualized using pygenomeviz.

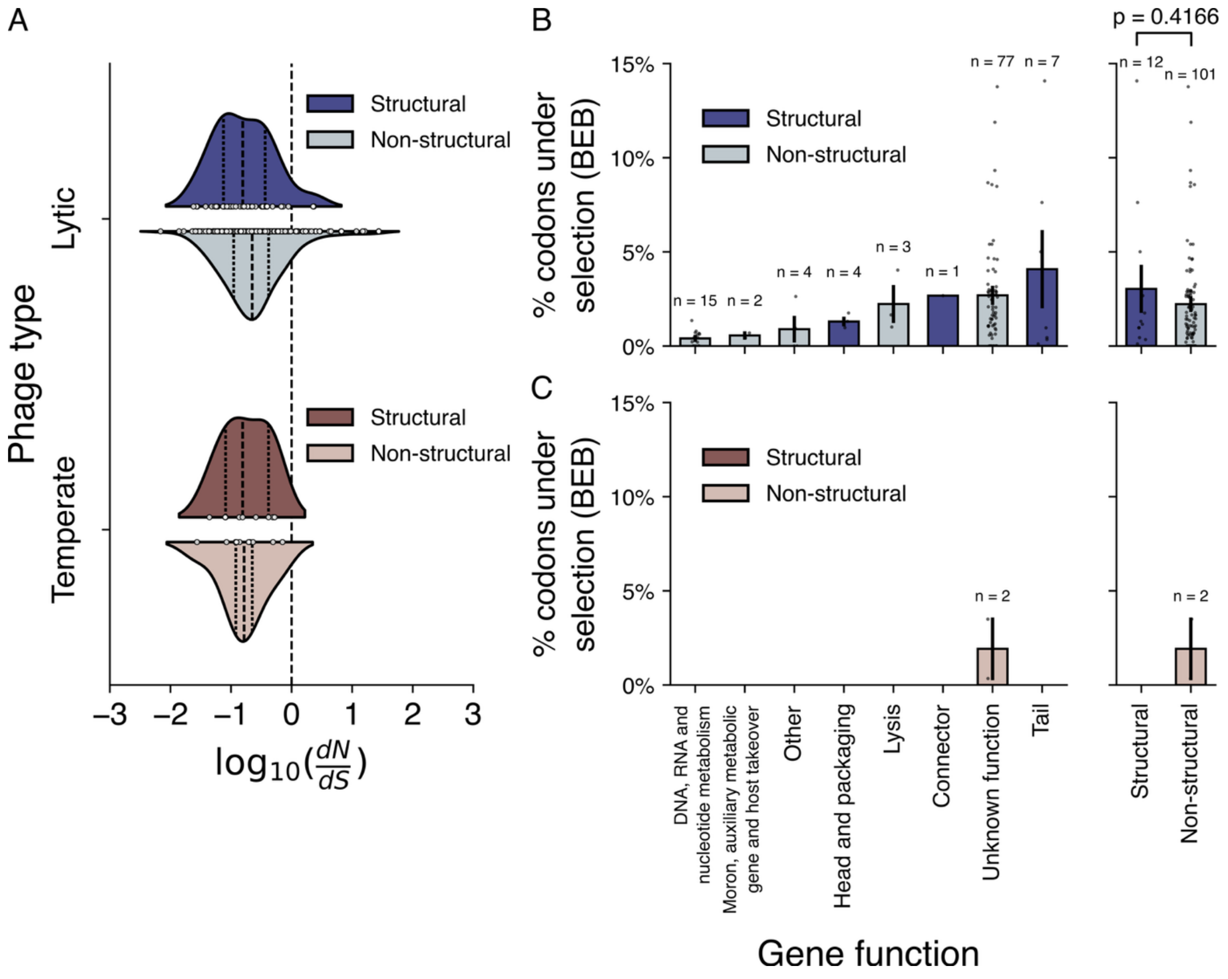

Figure S31. Analysis of positive selection in persistent phage genes. Selection for non-synonymous mutations was computed using the codeml functions of PAML using codon-alignments of single-copy orthologs in the lytic phage and prophage species found across multiple years. Inferences were performed using site-models M2a and M1a and informed by the species trees. **A.** The distribution of dN/dS values calculated using PAML are shown for core genes in the persistent lytic and prophage species. Each violinplot shows the distribution of log10(dN/dS), together with first quartile, median, and third quartile as internal dashed lines. Individual values are shown as points along the violinplot margin. Plots are split to show the distribution for structural (head, tail, and connector genes) and non-structural phage genes. The dashed vertical line shows where dN/dS is 1, implying neutrality. The significance calculations show the results of a one-sample Wilcoxon signed-rank one-sided test against a null hypothesis of a log10(dN/dS) of 1 to test for purifying selection. **B, C.** Genes under selection were filtered for using a likelihood ratio test between M2a and M1a site-models. Codons were considered under selection if the Bayes Empirical Bayes (BEB) probability that a site was under positive selection was greater than 80%. The number of codons under selection in each gene was normalized to the total length of the alignment. Error bars show mean +- SEM, and the significance calculation in panel B shows the output of a non-parametric Brunner-Munzel test. Individual data points are shown using overlaid dot plots.

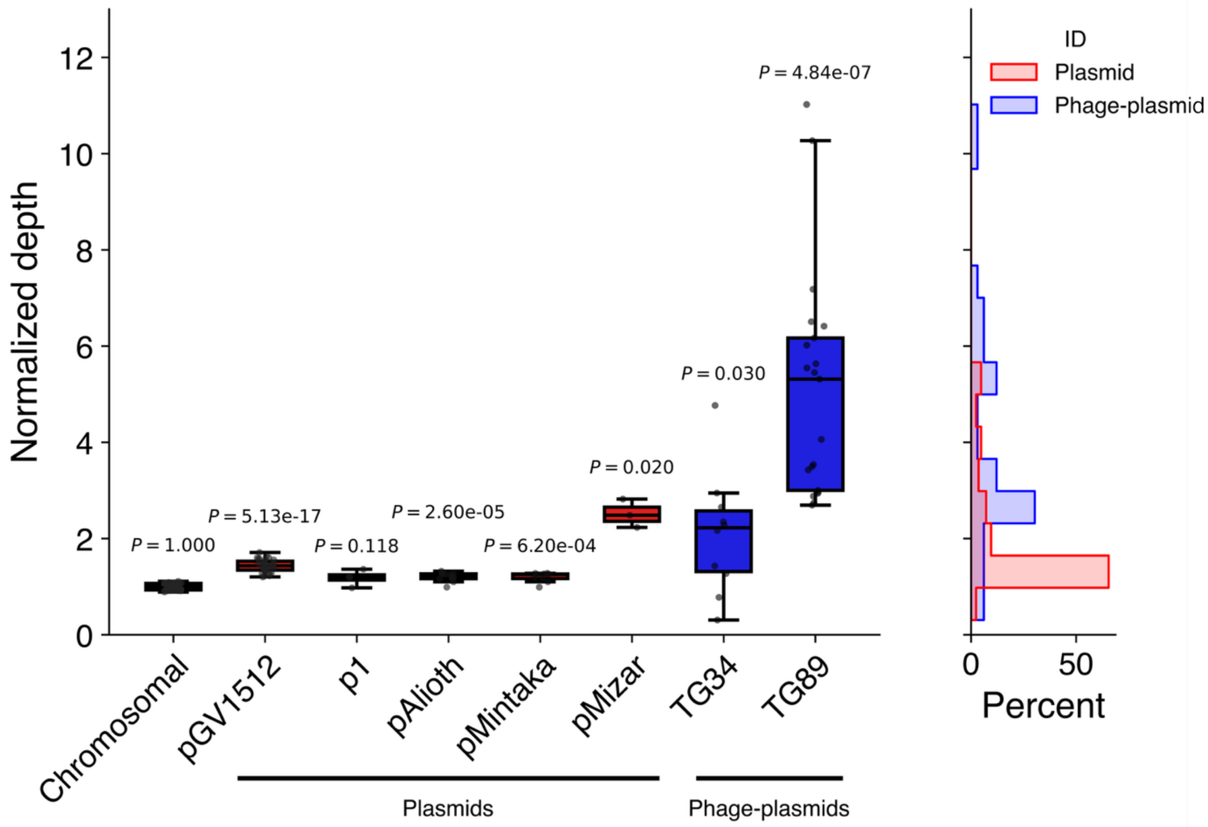

Figure S32. Relative short reads coverage of chromosome and plasmid in *V. crassostreae*. Illumina short reads of the 31 *V. crassostreae* strains containing phage-plasmids of TG34 and TG89 were remapped against their scaffolded assemblies to infer their relative copy number in the bacterial cell. For each strain, the depth of read coverage against each variety of contig was normalized to the mean coverage against chromosomes 1 and 2. Data are presented as boxplots (center line, median; box, 25th–75th percentiles; whiskers, 1.5 x interquartile range) with all individual points shown. Statistical significance was computed from a one-sample t-test with Benjamini-Hochberg control for false discovery rate against an expected coverage value of 1, to represent the expected coverage of a prophage integrated into the host chromosome. The histogram to the right shows the distribution of coverage depth for all plasmids and all phage-plasmids.

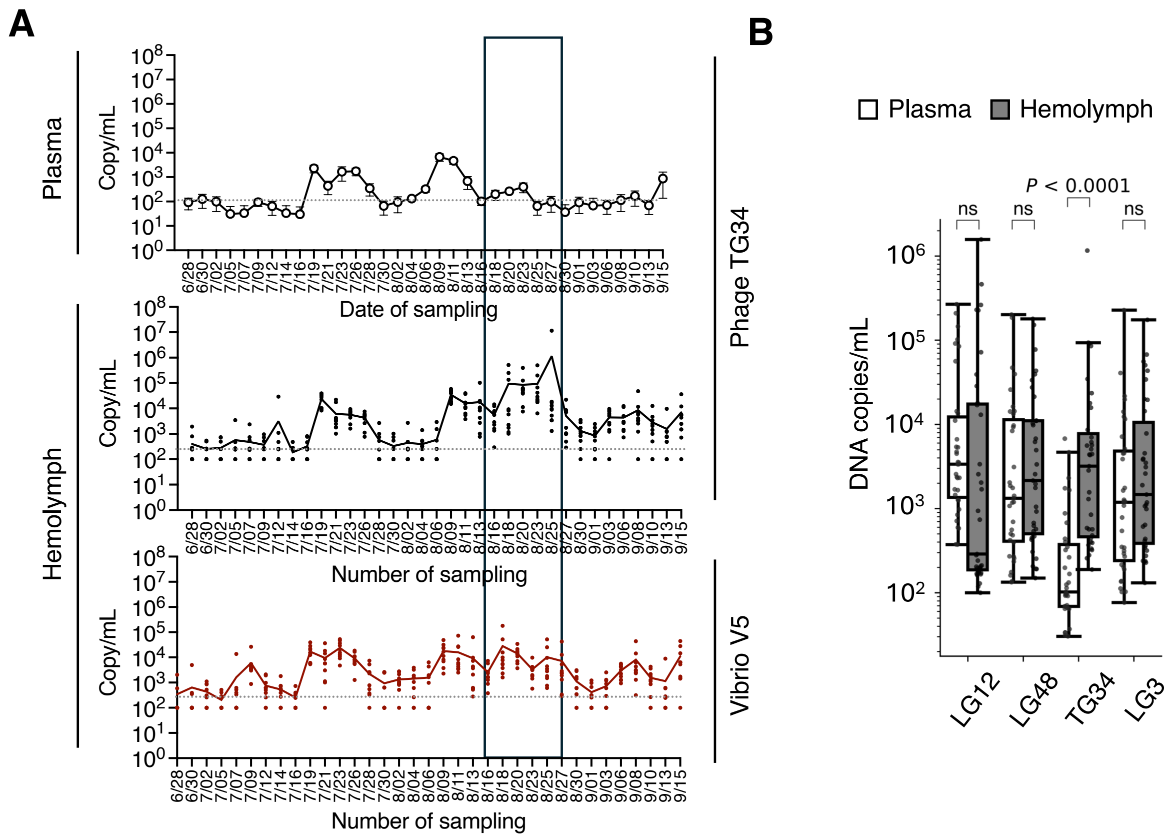

Figure S33. Induction and lysogeny of an active temperate phage in oyster hemolymph. A. ddPCR quantification of the temperate phage genus TG34 and vibrio clade V5 across the sampling season, measured in plasma (pooled from 90 oysters per date, four technical replicates) and hemolymph (10 individual oysters). The dotted line marks the quantification limit. Detection of phage DNA in plasma confirms activation, consistent with the recovery of lytic phages from the same genus (LG49, Figure 2A). From August 18 to 27 (inset), TG34 abundance spiked exclusively in hemolymph without a corresponding increase in V5, suggesting activation followed by lysogeny in other clades. This is consistent with the predicted host range of TG34, which includes clades V2 and V4 (Figure 5A). **B.** Most virulent phages (LG3, LG12, LG48) displayed comparable abundances in plasma and hemolymph. In contrast, TG34 reached significantly higher levels in hemolymph, supporting its temperate lifestyle. Statistical significance was tested using the two-sided Brunner-Munzel test with post-hoc correction for four comparisons. Individual data points are shown using overlaid dot plots.

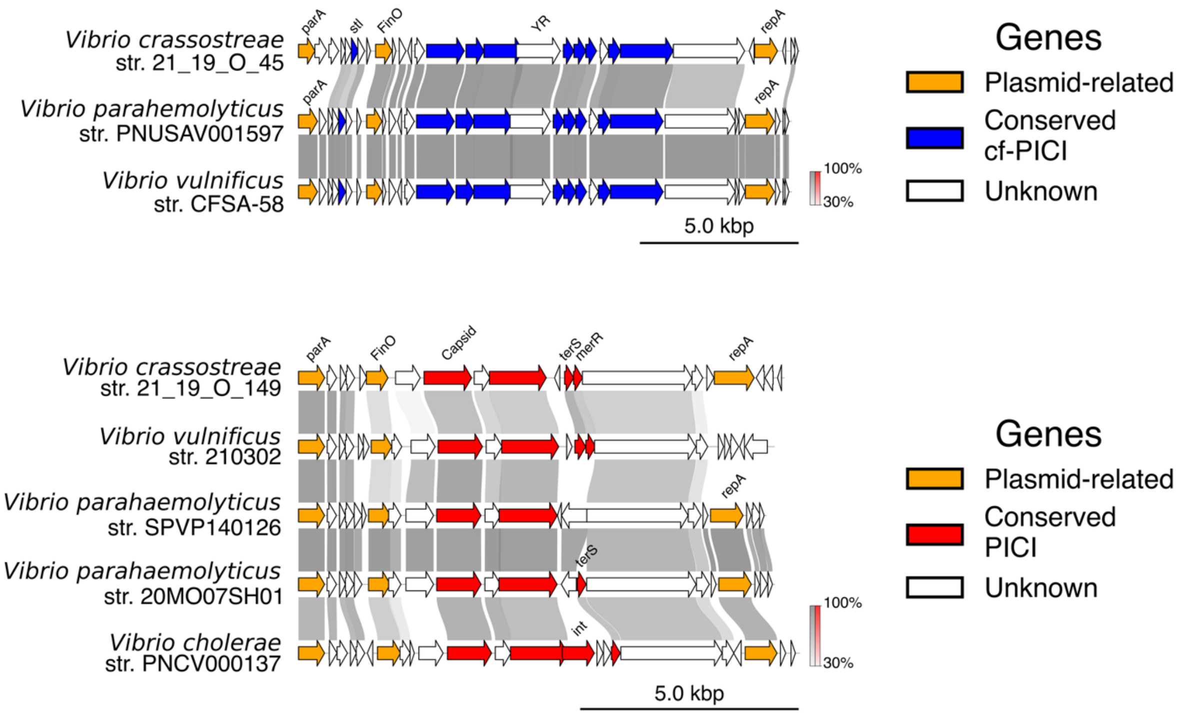

Figure S34. Plasmid-satellites are distributed widely across *Vibrio sp.* We screened for elements similar to the plasmid-satellites described in our dataset in public datasets by targeting assemblies with closely related capsid (or putative capsid modification protein; PICIs and PISPs) or parA gene sequences. Links between adjacent tracks show the percent identity of bi-directional best hits computed by MMseqs with a minimum identity of 30%. The sequence and synteny of the satellite modules are well-preserved in both the PISP and cf-PISP elements. Without exception, the homologous elements were assembled as contigs separate from the bacterial chromosomes which, together with their carriage of plasmid-like genes, strongly supports their identification as similar plasmid-satellites.
