# Supplementary material for: Complex temporal dynamics of phage-bacteria populations in an animal-associated marine system
Source: Nat Commun. 2026 Apr 4;17:4870. doi: 10.1038/s41467-026-71398-9 (PMC13230959; doi:10.1038/s41467-026-71398-9)
Supplement: Supplementary file 2 — Description of Additional Supplementary Files [file 41467_2026_71398_MOESM2_ESM.pdf]

Description of Additional Supplementary Files

File Name: Supplementary Data 1
Description: Lytic phages isolated on V. crassostreae, general features

File Name: Supplementary Data 2
Description: Auto-correlation values of vibrio and their phages in oysters

File Name: Supplementary Data 3
Description: Cross-correlation values between vibrio and their phages in oysters

File Name: Supplementary Data 4
Description: Vibrio crassostreae isolated in the present study general features

File Name: Supplementary Data 5
Description: Panstripe fitted parameters from pangenome presence/absence data and core genome phylogeny inferred using PanACoTA

File Name: Supplementary Data 6
Description: Panstripe comparisons with the models shown in Supplementary Data 5

File Name: Supplementary Data 7
Description: Regions of genomic plasticity in V. crassostreae, their MGE classification, and their gene contents

File Name: Supplementary Data 8
Description: Distribution of MGEs across niches

File Name: Supplementary Data 9
Description: Detailed significance testing of coding density between MGE types in V. crassostreae

File Name: Supplementary Data 10
Description: Predicted gene products on a representative hybrid assembled sequence of plasmid pGV

File Name: Supplementary Data 11
Description: Predicted gene products on a representative hybrid assembled sequence of plasmid pMintaka

File Name: Supplementary Data 12
Description: Predicted gene products on a representative hybrid assembled sequence of plasmid pAlioth

File Name: Supplementary Data 13
Description: Predicted gene products on a representative hybrid assembled sequence of plasmid pMizar

File Name: Supplementary Data 14
Description: Predicted gene products on a representative hybrid assembled sequence of plasmid p1

File Name: Supplementary Data 15
Description: Phages predicted from V. crassostreae sequence assemblies, general features

File Name: Supplementary Data 16
Description: Predicted Caudoviricetes prophages with genome size >25 kbp

File Name: Supplementary Data 17
Description: Prevalence of positive selection in single-copy ortholog genes assessed by PAML

File Name: Supplementary Data 18
Description: Primers used in this study
