# Supplementary material for: Complex temporal dynamics of phage-bacteria populations in an animal-associated marine system
Source: Nat Commun. 2026 Apr 4;17:4870. doi: 10.1038/s41467-026-71398-9 (PMC13230959; doi:10.1038/s41467-026-71398-9)
Supplement: Supplementary file 4 — Reporting Summary [file 41467_2026_71398_MOESM4_ESM.pdf]

nature portfolio

Corresponding author(s): LE ROUX Frédérique

Last updated by author(s): 2025/10/29

# Reporting Summary

Nature Portfolio wishes to improve the reproducibility of the work that we publish. This form provides structure for consistency and transparency in reporting. For further information on Nature Portfolio policies, see our Editorial Policies and the Editorial Policy Checklist.

## Statistics

For all statistical analyses, confirm that the following items are present in the figure legend, table legend, main text, or Methods section.

| n/a | Confirmed | |
|---|---|---|
| ☐ | ☒ | The exact sample size (*n*) for each experimental group/condition, given as a discrete number and unit of measurement |
| ☐ | ☒ | A statement on whether measurements were taken from distinct samples or whether the same sample was measured repeatedly |
| ☐ | ☒ | The statistical test(s) used AND whether they are one- or two-sided<br>*Only common tests should be described solely by name; describe more complex techniques in the Methods section.* |
| ☐ | ☒ | A description of all covariates tested |
| ☐ | ☒ | A description of any assumptions or corrections, such as tests of normality and adjustment for multiple comparisons |
| ☐ | ☒ | A full description of the statistical parameters including central tendency (e.g. means) or other basic estimates (e.g. regression coefficient) AND variation (e.g. standard deviation) or associated estimates of uncertainty (e.g. confidence intervals) |
| ☐ | ☒ | For null hypothesis testing, the test statistic (e.g. *F*, *t*, *r*) with confidence intervals, effect sizes, degrees of freedom and *P* value noted<br>*Give P values as exact values whenever suitable.* |
| ☐ | ☒ | For Bayesian analysis, information on the choice of priors and Markov chain Monte Carlo settings |
| ☒ | ☐ | For hierarchical and complex designs, identification of the appropriate level for tests and full reporting of outcomes |
| ☐ | ☒ | Estimates of effect sizes (e.g. Cohen's *d*, Pearson's *r*), indicating how they were calculated |

*Our web collection on statistics for biologists contains articles on many of the points above.*

## Software and code

Policy information about availability of computer code

| | |
|---|---|
| Data collection | Data was collected into Excel spreadsheets on Mac by Frédérique Le Roux |
| Data analysis | Software<br>All data collection and analyses were performed using publicly available, open-source software.<br>All software tools, packages, and version numbers are listed in the Methods section and include standard bioinformatic, phylogenetic, and statistical software.<br><br>Snippy v4.6.0: (https://github.com/tseemann/snippy) — SNP calling and variant mapping.<br><br>MAFFT v7.526 — multiple sequence alignments of proteins and coding sequences.<br><br>Unicycler v0.4.9 — hybrid assembly of Illumina and PacBio reads.<br><br>Trimmomatic v0.39 — read trimming and quality filtering.<br><br>SPAdes v3.15.2 — de novo genome and phage assemblies.<br><br>Bandage v0.9.0 — visualization of assembly graphs.<br><br>RagTag v2.1.0 with minimap2 v2.24–2.28 — genome scaffolding and read mapping. |

samtools v1.20 — read depth calculation, consensus generation, and coverage statistics.

fastp v0.24.0 — read cleaning before mapping.

bakta v1.9.2 (database v5.1) — automated bacterial genome annotation.

CONJscan v2.0.1 and MOBscan — identification and classification of conjugation systems and relaxases.

pharokka v1.7.3 (database v1.4.0) — phage genome annotation using Phanotate gene prediction.

HMMER (hmmsearch v3.4 / v3.3.2) — HMM-based gene identification.

SatelliteFinder v0.9 (with MacSyFinder v2.0) — prediction of phage satellites (PICI, cf-PICI, PLE, P4).

geNomad v1.8.1 — prophage detection and classification (database v1.7).

checkV v1.0.3 — quality control and completeness assessment of viral genomes.

ppanggolin v2.2.1 (panRGP module) — identification of genomic plasticity regions (RGPs).

PanACoTA v1.3.1-dev2 — pangenome construction and core-genome phylogeny.

IQ-TREE v2.3.6 / v2.0.6 — phylogenetic inference using maximum-likelihood methods.

panstripe v0.3.1 — phylogeny-aware pangenome analysis.

phylolm v2.6.5 and phytools v2.4-4 — phylogenetic comparative methods in R (v4.4.1).

VIRIDIC v1.1 — intergenomic similarity calculation and viral taxonomy.

BACPHLIP v0.9.6 — lysogeny prediction for phage genomes.

MMseqs2 v15-6f452 — protein clustering and homology searches.

prodigal v2.6.3 — gene prediction for plasmids and metagenomic sequences.

padloc v2.0.0 — detection of anti-phage defense systems.

BacMet2 (experimentally confirmed gene set), CARD v4.0.1, VFDB core dataset — identification of resistance and virulence genes via MMseqs2.

InterProScan and blastp (RefSeq release 229) — functional annotation of unknown proteins.

AlphaFold3 Server and FoldSeek (3Di/AA mode) — structural homology searches.

BEAST v2.6.3 — substitution rate inference under coalescent models.

PhiPack v1.0, Gubbins v3.4 — recombination detection and masking.

pyGenomeViz v1.4.1 — visualization of genomic synteny.

R (v4.4.1) — statistical analyses and figure generation (packages: ggraph 2.2.1, scipy, scikit_posthocs).

codeml under PAML 4.10.9 - Inference of dN/dS

Statistical analyses and figure generation were performed using R (v4.4.1) and Python (v3.10; packages: scipy, scikit_posthocs, astropy, statsmodels).

Code
Minor custom Python and R scripts were used for data parsing, replicon identification, wGRR calculation, and figure generation.
All custom scripts are publicly available on Zenodo (https://doi.org/10.5281/zenodo.18717761).
No proprietary software or custom algorithms were developed for this study.

For manuscripts utilizing custom algorithms or software that are central to the research but not yet described in published literature, software must be made available to editors and reviewers. We strongly encourage code deposition in a community repository (e.g. GitHub). See the Nature Portfolio guidelines for submitting code & software for further information.

## Data

Policy information about availability of data

All manuscripts must include a data availability statement. This statement should provide the following information, where applicable:

- Accession codes, unique identifiers, or web links for publicly available datasets
- A description of any restrictions on data availability
- For clinical datasets or third party data, please ensure that the statement adheres to our policy

All data supporting the findings of this study are publicly available. This includes:
Data availability
The sequenced genomes of the phages and Vibrio crassostreae isolated in the present study that have been deposited in the ENA data repository under bioprojects PRJEB81325 and PRJEB67885 respectively, with accession numbers listed in Supplementary Tables S1 and S4 respectively.

Collection of microorganisms
The phage and their vibrio hosts have been deposited in the following public collection: https://roscoff-culture-collection.org/ with accession numbers listed in Tables S1 and S4.

Source data are provided with this paper.
ddPCR data, isolation matrices, and environmental metadata (sampling date, temperature, salinity, mortality rate) are available as source data files with the article
All analyses were performed using publicly available, open-source software listed with version numbers in the Methods section.

## Research involving human participants, their data, or biological material

Policy information about studies with human participants or human data. See also policy information about sex, gender (identity/presentation), and sexual orientation and race, ethnicity and racism.

| | |
|---|---|
| Reporting on sex and gender | NA |
| Reporting on race, ethnicity, or other socially relevant groupings | NA |
| Population characteristics | NA |
| Recruitment | NA |
| Ethics oversight | NA |

Note that full information on the approval of the study protocol must also be provided in the manuscript.

# Field-specific reporting

Please select the one below that is the best fit for your research. If you are not sure, read the appropriate sections before making your selection.

☐ Life sciences ☐ Behavioural & social sciences ☒ Ecological, evolutionary & environmental sciences

For a reference copy of the document with all sections, see nature.com/documents/nr-reporting-summary-flat.pdf

# Ecological, evolutionary & environmental sciences study design

All studies must disclose on these points even when the disclosure is negative.

| | |
|---|---|
| Study description | This study investigates the ecological and evolutionary dynamics of phage–host interactions in a natural marine community composed of the oyster pathogen Vibrio crassostreae, its virulent and temperate bacteriophages, and associated mobile genetic elements.<br>Longitudinal sampling was conducted at an oyster farm in the Bay of Brest (France) three times per week from June to September 2021, covering the full period of an oyster mortality outbreak.<br>The study integrates field ecology, quantitative infection assays, genome sequencing, and comparative genomics to link phage predation patterns with bacterial population structure, mobile element diversity, and viral persistence over time.<br>Analyses include the isolation and genomic characterization of 447 V. crassostreae strains and 1,331 phages, the prediction and classification of prophages, plasmids, and phage satellites, and the inference of evolutionary rates and phylogenetic relationships.<br>Together, these data reveal how ecological constraints shape both viral–host lineage stability and mobile element diversification in the marine environment. |

Research sample

The research focused on marine bacteria of the Vibrio crassostreae species complex, their associated bacteriophages, and mobile genetic elements (prophages, plasmids, and phage satellites) within a natural coastal ecosystem.
Samples were collected from an oyster farm in the Bay of Brest, France, between June 28 and September 15, 2021, during an ongoing oyster mortality outbreak.
The study analyzed:
447 genome-sequenced Vibrio crassostreae isolates out of 512 confirmed strains (selected to represent all major clades and sampling dates);
1,331 purified lytic phages, isolated from seawater and oyster hemolymph using a reference collection of 153 V. crassostreae hosts;
Predicted prophages, plasmids, and satellites identified across 605 bacterial genomes through comparative genomics.
These biological samples represent a natural host–phage–environment continuum, providing sufficient replication across time and ecological compartments (oysters vs. seawater) to assess both short-term and persistent virus–host associations.

Sampling strategy

Sampling was designed to capture temporal and ecological variation in Vibrio crassostreae and its phages during a natural oyster mortality event.
From June 28 to September 15, 2021, samples were collected three times per week from a single oyster farm in the Bay of Brest (Pointe du Château, France), corresponding to a 12-week time series encompassing the full period of elevated seawater temperatures (>16 °C) associated with oyster disease.
At each time point:
100 live oysters were collected, and their hemolymph was processed both for bacterial isolation and viral concentration;
10 L of seawater were filtered into size fractions (60, 5, 1, 0.2 µm) to recover microbial and viral communities.
Sampling targeted distinct ecological compartments (oysters vs. seawater) and was replicated 35 times across the season to ensure temporal resolution.
Specific Pathogen-Free (SPF) oysters were deployed weekly to standardize host exposure conditions and mortality monitoring.
From each sampling, random subsamples of bacterial colonies (≈ 96 per source per date) were screened to identify V. crassostreae isolates representing the diversity present in the environment.
This design ensured adequate statistical power to detect within- and between-date variation in phage predation and to link ecological dynamics with genomic diversification.

Data collection

Data were collected through coordinated field sampling, laboratory isolation, and genomic analyses.
From each of the 35 sampling dates (June–September 2021), seawater and oyster hemolymph were processed for bacterial and viral isolation.
Bacterial isolates were cultured on TCBS agar, screened by multiplex PCR, and identified as Vibrio crassostreae through zrgB gene sequencing and phylogenetic assignment.
Viral concentrates were obtained by iron chloride flocculation from seawater and by filtration of oyster plasma, followed by plaque assays using a reference panel of 153 V. crassostreae strains to detect phage predation.
Distinct plaque morphotypes were purified to generate a phage collection of 1,331 isolates.
DNA was extracted from bacteria, phages, and environmental fractions for Illumina short-read sequencing, with selected strains and phages; 20 vibrio were  also sequenced by PacBio long reads.
Environmental DNA (seawater, oyster hemolymph, hemomicrobiota) was archived for ddPCR.
All genomic data were processed through standardized bioinformatic workflows for quality control, assembly, and annotation, as detailed in the Methods section.
Field metadata (sampling date, temperature, Salinity, oyster mortalitys were recorded systematically during collection.

Timing and spatial scale

Sampling was conducted over a 12-week period, from June 28 to September 15, 2021, at an oyster farm in the Bay of Brest (Pointe du Château, 48° 20′ 06.19″ N, 4° 19′ 06.37″ W), France.
The campaign began when seawater temperatures exceeded 16 °C—a threshold associated with oyster disease outbreaks—and continued throughout the full duration of the mortality episode.
Samples were collected three times per week (35 sampling dates in total), providing high-frequency temporal resolution across the summer season.
Spatially, all samples were taken from a single, well-characterized aquaculture site to minimize environmental heterogeneity, while two ecological compartments—seawater and oysters—were systematically sampled to capture within-site variation in microbial and viral communities.
This design allowed detection of fine-scale temporal dynamics in phage–host interactions and comparisons between host-associated and free-living microbial populations within the same marine ecosystem.

Data exclusions

Data exclusions were applied only based on objective quality control criteria.
Among the 512 Vibrio crassostreae isolates confirmed by PCR, 59 strains (11%) were excluded from genomic analyses because they represented divergent lineages or yielded incomplete or low-quality sequencing data.
Similarly, a small number of phage genomes were excluded when assemblies were fragmented, contaminated, or lacked the expected viral hallmark genes as identified by geNomad or checkV.
For environmental and ddPCR datasets, only wells or samples meeting minimum quality thresholds (e.g., ≥10,000 droplets per well for ddPCR, or sufficient DNA yield for sequencing) were retained.
All exclusion criteria were predefined and applied consistently across datasets; no data were removed to alter statistical outcomes or interpretations.

Reproducibility

Experimental and analytical procedures were designed to ensure robustness and reproducibility, given the logistical and biological constraints of field-based ecological sampling.
Phage infection screening (Figures 1 and 3A) was performed once per sampling date, but the dataset includes 35 independent sampling dates across the 2021 season, each representing a biologically independent replicate in time.
This design captures reproducible ecological patterns rather than technical variation, and consistent infection structures were observed across the time series.
Droplet digital PCR (ddPCR) analyses (Figure 3B) were also performed once per target due to cost constraints, but each time point included 10 individually analyzed oysters, providing biological replication within each date.
All phage isolates were purified through up to three rounds of plaque isolation to ensure clonality and stability.
Genome sequencing and bioinformatic analyses followed standardized, version-controlled pipelines, producing consistent results across datasets.
Together, these approaches ensure that the key findings are reproducible through independent biological replication (across time and individuals), even when technical replication was limited by the scale of the study.

Randomization

Randomization was incorporated at multiple stages of the study to minimize sampling and analytical bias.
During each of the 35 field sampling dates, oysters were randomly collected from the deployed cohort (≤50% mortality) without prior selection based on phenotype or size.
For bacterial isolation, approximately 96 colonies per sample type (seawater and hemolymph) were randomly picked from TCBS plates to ensure an unbiased representation of the culturable Vibrio population.
Phage isolation used all available host strains from the reference Vibrio crassostreae collection, with no pre-selection for infection phenotype.
Primer and probe validation steps were applied during ddPCR assay design to avoid systematic bias toward specific loci or clades.
No formal treatment or control groups were used, as this study describes natural ecological variation rather than a manipulative experiment; however, the consistent randomization of biological material and the replication across time points provide statistical independence among samples.

Blinding

Blinding was not applicable to this study, as it involved observational and culture-based analyses of environmental samples rather than experimental treatments or subjective scoring.
Sample identities (date, source, host clade) were automatically recorded during collection and processing but were not used to influence data acquisition or interpretation.
All sequencing, bioinformatic processing, and statistical analyses were conducted using automated, reproducible pipelines, ensuring objective handling of the data.
Phage screen and ddPCR outputs were quantified based on numerical readouts (plaque counts, droplet fluorescence), eliminating observer bias.
Figures and analyses were independently verified by multiple co-authors to confirm consistency and accuracy of results.

Did the study involve field work? ☒ Yes ☐ No

## Field work, collection and transport

Field conditions

Fieldwork was conducted at a commercial oyster farm located in the Bay of Brest, France (Pointe du Château, 48° 20′ 06.19″ N, 4° 19′ 06.37″ W), under natural marine environmental conditions.
Sampling took place three times per week from June 28 to September 15, 2021, during the summer period when seawater temperatures exceeded 16 °C, the threshold associated with oyster mortality events.
Environmental parameters (temperature, salinity, and oyster mortality rate) were recorded at each visit.
The site experienced no experimental manipulation; all samples were collected under normal aquaculture operations and reflect natural ecological variability.
Weather and tidal conditions were typical for the season and did not affect sampling frequency or accessibility.
The field campaign coincided with an ongoing episode of oyster mortality, providing an ideal context to study host–phage dynamics under ecologically relevant stress conditions.

Location

All fieldwork was carried out at a single, well-characterized oyster farming site in the Bay of Brest, Brittany, France — specifically at Pointe du Château (48° 20′ 06.19″ N, 4° 19′ 06.37″ W).
This coastal site is part of a semi-enclosed bay influenced by both oceanic and estuarine water masses, providing stable yet ecologically diverse marine conditions favorable for studying host–phage interactions.
The farm hosts Pacific oysters (Crassostrea gigas) cultivated under standard aquaculture practices and has a documented history of recurrent Vibrio-associated mortality events, making it a natural model system for investigating bacterial and viral community dynamics.
All collections were performed within the same site to ensure environmental consistency and to allow high-resolution temporal comparison of seawater and oyster-associated microbial populations.

Access & import/export

Access to the field site was granted by the local oyster farm manager in coordination with regional aquaculture authorities.
Sampling was conducted in compliance with French and European regulations governing environmental monitoring and aquaculture research.

Because the study involved non-experimental collection of seawater and oyster samples from an established commercial site, no specific ethical approval or collection permit was required beyond farm owner consent.
All oyster batches used were Specific Pathogen-Free (SPF) juveniles deployed under standard aquaculture protocols, and no manipulation of live vertebrates or protected species occurred.
Transport and handling of biological material complied with biosafety and biosecurity guidelines established by Ifremer and Roscoff marine station. The subsequent import of Vibrio strains and bacteriophages to Canada was performed under the required import permits issued by the Canadian Food Inspection Agency (CFIA), ensuring full compliance with national biosafety and quarantine regulations.

Disturbance

Field activities caused no measurable disturbance to the environment or to aquaculture operations.
Sampling consisted of collecting limited volumes of seawater (10 L per visit) and a small subset of oysters (100 individuals) from ongoing commercial batches already used for mortality monitoring.
No experimental manipulation, habitat alteration, or introduction of foreign material occurred during sampling.
All collections followed routine aquaculture handling procedures, and the removal of specimens represented a negligible fraction of the total stock.
Thus, the study had no ecological or operational impact on the oyster farm or surrounding marine environment.

# Reporting for specific materials, systems and methods

We require information from authors about some types of materials, experimental systems and methods used in many studies. Here, indicate whether each material, system or method listed is relevant to your study. If you are not sure if a list item applies to your research, read the appropriate section before selecting a response.

## Materials & experimental systems

| n/a | Involved in the study | |
|---|---|---|
| ☒ | ☐ | Antibodies |
| ☒ | ☐ | Eukaryotic cell lines |
| ☒ | ☐ | Palaeontology and archaeology |
| ☐ | ☒ | Animals and other organisms |
| ☒ | ☐ | Clinical data |
| ☒ | ☐ | Dual use research of concern |
| ☒ | ☐ | Plants |

## Methods

| n/a | Involved in the study | |
|---|---|---|
| ☒ | ☐ | ChIP-seq |
| ☒ | ☐ | Flow cytometry |
| ☒ | ☐ | MRI-based neuroimaging |

## Animals and other research organisms

Policy information about studies involving animals; ARRIVE guidelines recommended for reporting animal research, and Sex and Gender in Research

Laboratory animals

No vertebrate laboratory animals were used in this study.
The research involved Pacific oysters (Crassostrea gigas), a marine invertebrate species not covered by animal welfare legislation.
All oysters were Specific Pathogen-Free (SPF) juveniles, reared under standard aquaculture conditions by licensed producers before deployment in the Bay of Brest.
Handling and sampling of oysters were performed following Ifremer's animal welfare and biosecurity guidelines to minimize stress and ensure safe disposal of biological material.
No procedures involving pain, anesthesia, or experimental infection were conducted; oysters were collected solely for ecological and microbiological analyses of naturally occurring mortality events.

Wild animals

No vertebrate or protected wild animals were involved in this study.
Sampling targeted Pacific oysters (Crassostrea gigas) cultivated in an aquaculture facility and the associated microbial and viral communities present in surrounding seawater.
Although the study investigated naturally occurring marine microorganisms (including Vibrio crassostreae and their bacteriophages) from the environment, no disturbance, manipulation, or depletion of wild populations occurred.
All collections complied with French environmental and aquaculture regulations and were carried out under farm owner consent.
The work thus represents environmental microbiological monitoring, not wildlife experimentation.

Reporting on sex

Oysters were diploid but not sexted to determine male or female.

Field-collected samples

Field-collected samples consisted of seawater and oysters (Crassostrea gigas) obtained from a commercial aquaculture site in the Bay of Brest, France.
Sampling occurred three times per week between June 28 and September 15, 2021, during a natural oyster mortality episode.
At each date, 10 L of seawater were collected and 100 live oysters (≤50% mortality) were sampled from the deployed cohort.

Oysters were transported to the laboratory in insulated containers at ambient seawater temperature and processed within a few hours of collection. Oysters were killed by shucking them with an oyster knife.
Hemolymph was extracted  using a syringe from adductor muscle and both bacterial and viral fractions were isolated for downstream genomic and microbiological analyses.
Seawater samples were size-fractionated by filtration (60, 5, 1, and 0.2 µm) to separate microbial and viral communities.
All samples were handled using sterile equipment and preserved appropriately (e.g., −80°C for DNA extraction, 4°C for viral concentrates) to ensure data integrity and reproducibility.

Ethics oversight

No ethical approval was required in accordance to Europe animal welfare legislation which does not include bivalve mollusks

Note that full information on the approval of the study protocol must also be provided in the manuscript.

## Plants

Seed stocks

NA

Novel plant genotypes

NA

Authentication

NA
